# Decoding spatiotemporal fibrotic and cellular immunosuppression of therapeutic T cells in live pancreatic ductal adenocarcinoma

**DOI:** 10.1038/s44320-026-00243-4

**Published:** 2026-09-10

**Authors:** Guhan Qian, Hongrong Zhang, Ingunn M Stromnes, Kevin W Eliceiri, Paolo P Provenzano

**Affiliations:** 1 Department of Biomedical Engineering, University of Minnesota, Minneapolis, MN USA; 2 Center for Multiparametric Imaging of Tumor Immune Microenvironments, University of Minnesota, Minneaplis, MN and University of Wisconsin-Madison, Madison, WI USA; 3 Department of Microbiology and Immunology, University of Minnesota, Minneapolis, MN USA; 4 Center for Immunology, University of Minnesota, Minneapolis, MN USA; 5 Center for Genome Engineering, University of Minnesota, Minneapolis, MN USA; 6 Masonic Cancer Center, University of Minnesota, Minneapolis, MN USA; 7 Center for Quantitative Cell Imaging, University of Wisconsin-Madison, Madison, WI USA; 8 Department of Medical Physics, University of Wisconsin-Madison, Madison, WI USA; 9 Department of Medicine, Division of Hematology, Oncology, and Transplantation, University of Minnesota, Minneapolis, MN USA

**Keywords:** Cancer, Immunology

## Abstract

Pancreatic ductal adenocarcinoma (PDA) is profoundly immunosuppressive. To help define this behavior, we present integrated experimental and computational frameworks to elucidate therapeutic T cell dynamics. Through the development of TME-CARTographer (TME-CART), a computational pipeline integrating high-dimensional data, graph theory, behavior analysis, and deep learning (DL), we present quantitative insights on 4D T cell-TME interactions in live PDA tumors. Mapping physical immunosuppression demonstrates that collagen fiber architectures direct migration while concomitantly limiting off-axis movement, creating immune exclusion zones. Expanding these findings, we establish that the collagen matrix harbors and spatially organizes immunosuppressive myeloid cells to serve as cooperative co-modulators of T cell behaviors, including migration, sampling, repulsion, and sequestration. Consistent with these findings, DL defines both linear and nonlinear collagen matrix and cellular neighborhood interactions as drivers of T cell behavior. The TME-CART DL framework also accurately predicts shifts in immunosuppression following depletion of myeloid cells. Overall, we identify synergistic barriers impeding anti-tumor T cell behaviors and present TME-CART as a discovery platform for interpreting complex 4D data to enhance the understanding and design of immunotherapies.

## Synopsis

Solid tumors such as pancreatic cancer resist T cell therapy. This study integrates in vivo and ex vivo perturbations, multiphoton microscopy of live tumor tissue, quantitative modeling, and deep learning to reveal stromal and cellular features that suppress therapeutic T cell movement and localization.

- Collagen networks in pancreatic cancer act as high-affinity “highways” that confine T cells to pre-defined paths, create physical immune exclusion zones, and limit tumor sampling through migration anisotropy.
- Immunosuppressive CD11b+ myeloid cells (TAMs/MDSCs) act as roadblocks that fragment infiltration routes, increase forced path tortuosity, and drive T cell sequestration.
- Deep learning with *post hoc* explanations (SHAP, LIME, 1- and 2-variable PDPs) resolves nonlinear cell-cell and cell-ECM interactions driving antigen-driven T cell behaviors and PD-1/PD-L1-mediated T cell suppression.
- Depleting myeloid cells relieves suppression and enhances therapeutic T cell dispersal and tumor sampling, as predicted by TME-CART and confirmed by pharmacologic depletion in vivo and ex vivo.

## Abstract (web summary)

Solid tumors such as pancreatic cancer resist T cell therapy. This study integrates in vivo and ex vivo perturbations, multiphoton microscopy of live tumor tissue, quantitative modeling, and deep learning to reveal stromal and cellular features that suppress therapeutic T cell movement and localization.

## Graphical abstract

**Figure:**
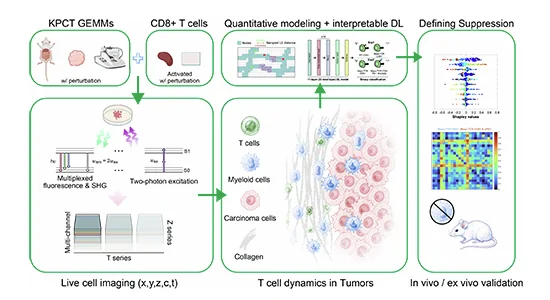

## Introduction

Despite recent improvements in all-stage 5-year survival rates, pancreatic ductal adenocarcinoma (PDA) remains an exceedingly lethal malignancy with a ~13% five-year survival rate in all stages and only ~3% in patients with distant metastasis (2025; Sohal et al, 2020). Effective treatment options are limited, thus there remains a desperate need for alternative therapeutic approaches. In this context, T cell-based therapies have emerged as a promising candidate to combat disease.

Immunotherapies with immune checkpoint blockade (ICB) or infusion of genetically modified T cells are producing remarkable clinical responses in some advanced hematologic malignancies and a small subset of solid tumors, such as melanoma (Carlino et al, 2021; Lim and June, 2017; Ribas et al, 2016; Ribas and Wolchok, 2018). However, outside of rare cases (Zhao et al, 2022), or following focused disruption of stromal structures and signaling (Feig et al, 2013; Jiang et al, 2016; Li et al, 2018; Morrison et al, 2020), most pancreatic cancers are resistant to ICB, e.g. (Brahmer et al, 2012; Feig et al, 2013; Jiang et al, 2016; Lee et al, 2016; Royal et al, 2010). This is due to multiple levels of immunosuppression from fibrotic and myeloid cell-dense tumor microenvironments and the imperfect capacity for T cells to specifically identify and eliminate target cells prior to exhaustion (Beatty et al, 2011; Elahi-Gedwillo et al, 2019; Jiang et al, 2016; Stromnes et al, 2014). Indeed, endogenous T cells can become trapped in solid tumor stroma, failing to access tumor cells (Hartmann et al, 2014; Jiang et al, 2016; Lou et al, 2025). Yet, in order to be effective, anti-tumor T cells must be in extremely close proximity or have direct interactions with cancer cells that they are primed to target, and they must navigate the robust spatial and temporal heterogeneity within tumors. Likewise, the presence of immunosuppressive myeloid cells such as tumor-associated macrophages (TAMs) and myeloid-derived suppressor cells (MDSCs) is robust in PDA, and approaches to reduce these populations have been shown to enhance anti-tumor immunity (Beatty et al, 2015; Dixit et al, 2022; Panni et al, 2019; Stromnes et al, 2014). Thus, while it has become clear that fibrous extracellular matrix (ECM) and immunosuppressive myeloid cells are known regulators of anti-tumor immune function, our understanding of their local spatiotemporal impact in TMEs remains limited.

Interestingly, in a genetically engineered mouse model predictive of therapeutic response in PDA patients, tumor-reactive engineered T cells carrying a cloned mesothelin-specific T-cell receptor (TCR: high-affinity Msln_406-414_:H2D^b^-specific TCR clone 1045) infiltrate PDA more effectively than endogenous T cells and significantly prolong survival (Stromnes et al, 2014b; Stromnes et al, 2015). Yet, they did not sample the entire tumor volume or result in complete responses, suggesting that further T cell enhancements are needed. Thus, defining T cell and TME features that dictate successful or unsuccessful effector-to-target interactions can provide design criteria for identifying strategies to further enhance key T cell behaviors in solid tumors and will inform stromal and cell engineering strategies for patient treatment. Here, within live tumors, we image T cells with multiplexed multiphoton microscopy. In the resulting images, we then dissect T cell dynamics using TME-CARTographer (TME-CART), a computational discovery pipeline enabled by novel algorithms for quantitative analysis to decode functional immunosuppression mediated by the stromal ECM and immunosuppressive myeloid cells. This incorporates an interpretable deep learning platform trained from a 23-dimensional feature space that is derived from microscopy data. Using local and global black-box explanations, we present and rank univariate and multivariate factors within the TME that regulate T cell behavior and functional immunosuppression. With our fully developed platform, we computationally predict that reducing myeloid-mediated immunosuppression effectively enhances T cell dispersal and promotes tumor sampling. Thus, our integrated data analysis platform offers a robust, quantitative analysis and screening tool for designing and testing combinatorial therapeutic strategies, such as cell-based therapies, stroma-targeting therapy, and immune modulators, aimed at optimizing engineering criteria for next-generation cell-based immunotherapies in solid tumors.

## Results

### TME-CART elucidates spatiotemporal immunosuppression of T cell dynamics in PDA

**Figure 1:**
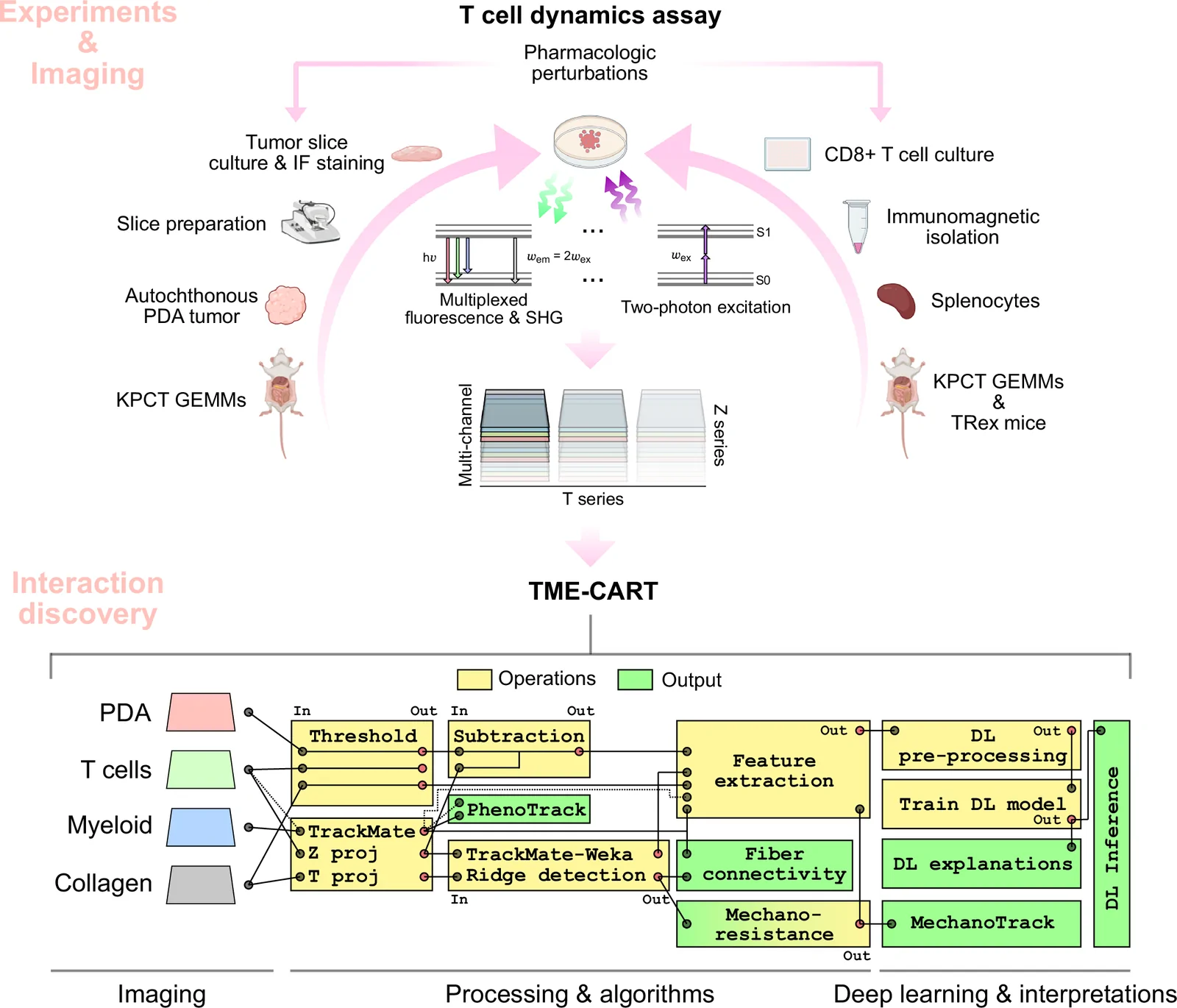
A pipeline that combines tumor models, imaging, and a computational discovery pipeline. TME-CARTographer (TME-CART) uses novel algorithms and deep learning with post hoc interpretations to unveil the intricate spatiotemporal behaviors and interactions of engineered therapeutic T cells and the ECM in the highly immunosuppressive PDA TME.

T cell migration and interaction with target cells have been investigated in tumor-derived organoid systems (Dekkers et al, 2022; Dijkstra et al, 2018; Peranzoni et al, 2018; Tsai et al, 2018) and solid tumors (Hartmann et al, 2014; Salmon et al, 2012; Tabdanov et al, 2021; You et al, 2021). However, our understanding of factors that direct and limit T cell movement in complex solid TME remains limited. We previously established a platform (Rodriguez-Merced et al, 2023; Tabdanov et al, 2021) that evaluates the migration of T cells in the autochthonous PDA tumor tissue from the *Kras*^*LSLG12D/+*^*; p53*^*LSL-R172H/+*^*; Pdx-1-Cre* (*KPC*) mouse model that faithfully replicates the human disease, including the stromal response associated with PDA (Hingorani et al, 2005; Provenzano et al, 2012) and provides an excellent tool to evaluate the influence of key stromal components, such as the dense collagen networks and robust levels of immunosuppressive myeloid cells associated with PDA. This system preserves the structural integrity of the TME, providing a faithful platform for examining T cell dynamics (Rodriguez-Merced et al, 2023). Here, along with utilizing fluorescent reporter for carcinoma cells, i.e., *KPC; ROSA26*^*LSL-tdTomato/+*^ (*KPCT*), we build upon this protocol by further incorporating live-cell immunofluorescence (IF) staining to visualize specific stromal populations of interest. Using multiphoton laser-scanning microscopy (MPLSM) to generate multiphoton excitation (MPE) of fluorescence and second harmonic generation (SHG) of fibrillar ECM, engineered T cells are tracked throughout the tumor volume across space and time, capturing spatiotemporal information on T cell dynamics in relation to TME features. To uncover and analyze latent T cell behaviors in the TME that influence T cell movement, we present TME-CART (Fig. 1), a computational discovery pipeline for processing spatiotemporal imaging data that characterizes the physical and cellular immunosuppression of the PDA TME and extracts the dynamic behaviors of T cells and the interplay between T cells and their local neighborhoods. By integrating biological experiments, imaging, and computational analysis, we developed a first-of-its-kind workflow, which is described and applied in the following sections. This discovery and screening platform for cell-based therapy helps establish engineering design criteria that can inform stromal manipulation and/or cell engineering strategies in solid tumors.

### A fibrotic immunosuppressive index to capture ECM-mediated immune exclusion zones

**Figure 2:**
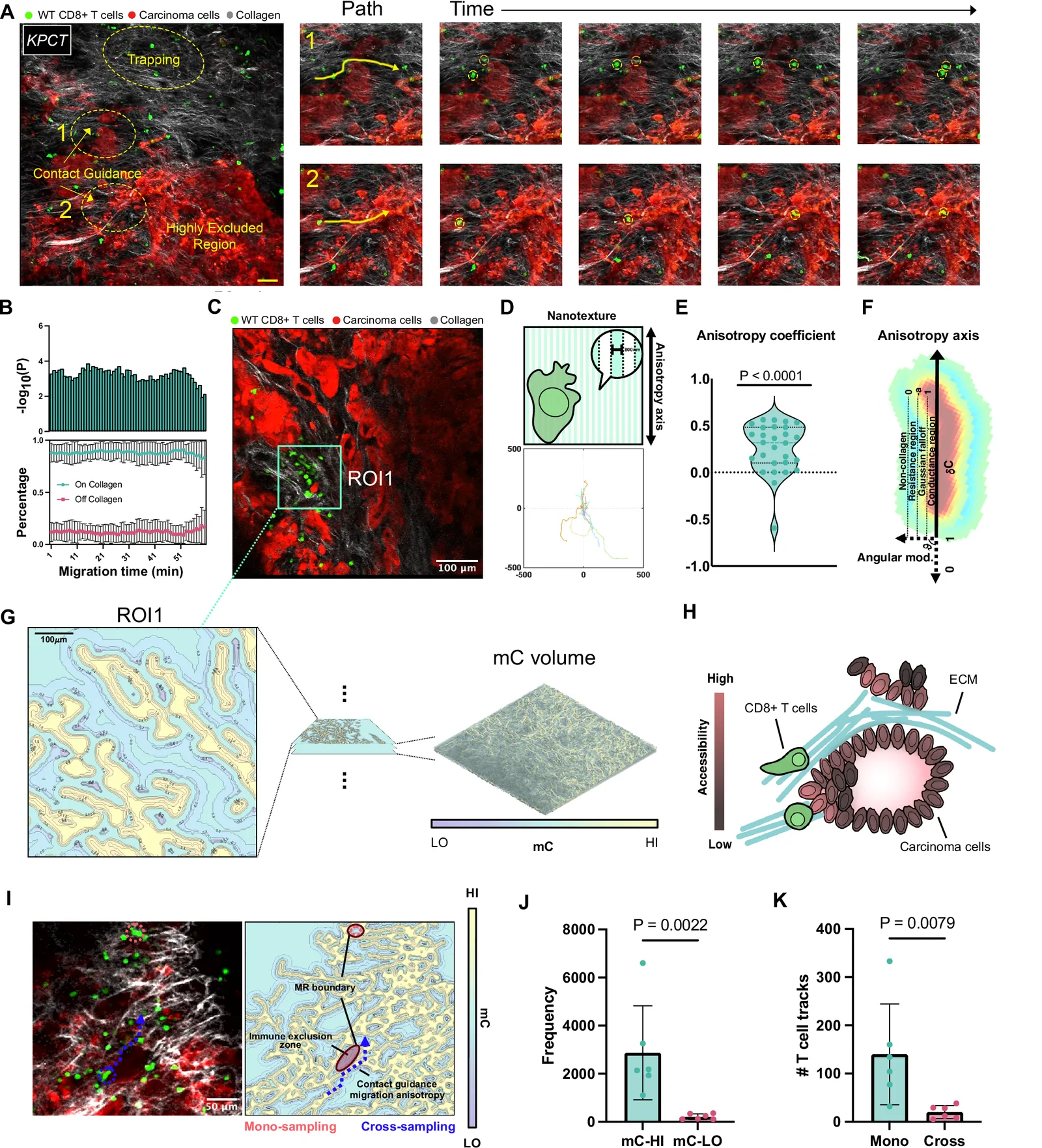
Defining a fibrotic immunosuppressive index to capture ECM-mediated immune exclusion regions. (**A**) MPLSM (combined MPE and SHG) time-lapse of WT CD8 + T cells (green), carcinoma cells (red), and collagen (gray) in a live PDA *KPCT* tumor slice demonstrate heterogeneous T cell behaviors due to ECM architecture. *Path 1 = upper yellow arrow showing T cell path*; yellow and orange circles highlight examples of T cells as they move along collagen fiber networks through contact guidance to move past carcinoma regions. *Path 2 = lower yellow arrow showing T cell path*; with adjacent panels with yellow circle highlighting a T cell moving into carcinoma-rich regions. Note, the first time series image matches the path image. (**B**) Object-based colocalization of WT CD8 + T cells (*n* = 618, multiple unpaired t tests; *p* values shown in figure) and collagen signal reveals that T cells and collagen consistently remain spatially colocalized throughout the observation period. (**C**) MPLSM series showing WT CD8 + T cells (green) in PDA tumor slice demonstrates increased clustering propensity in collagen-dominant neighborhoods. (**D**) T cells exhibit directionally biased movement on anisotropic nanotexture and T cell tracks on nanotexture. (**E**) Migration anisotropy coefficients (−1 – perfectly perpendicular motility; 0 – non-biased motility; 1 – perfectly parallel to nanotextures motility) were extracted from the tracked T cells (*n* = 28) with a median coefficient of 0.32 (*P* < 0.0001 *n* = 28, one-sample *t* test). (**F**) The MechanoTrack algorithm enables computational mapping of ECM mechanoresistance (mR) and mechanoconductance (mC). (**G**) Mapping mC in a live tumor slice quantitatively illustrates the topology of physical immunosuppression. (**H**) A model of immune exclusion explained by differential accessibility induced by non-uniform collagen architecture. (**I**) Illustrations of T cell sampling mechanisms reveal that migration anisotropy constrains T cell sampling within the same mR region (mono-sampling, red), rather than sampling across different mR regions (cross-sampling, blue). (**J**) T cells predominantly traversed high-mC regions (*P* = 0.0022, *n* = 6, unpaired *t* test). (**K**) T cells rarely cross-sample between high- and low-mC areas and prefer mono-sampling (*P* = 0.0079, *n* = 6, unpaired *t* test). Error bars represent standard deviation. Source data are available online for this figure.

Collagen is abundantly observed in PDA (Elahi-Gedwillo et al, 2019; Olive et al, 2009; Provenzano et al, 2012). Yet, the role of collagen in PDA progression remains debated, particularly when levels are manipulated prior to the onset of PDA (Chauhan et al, 2013; Elahi-Gedwillo et al, 2019; Ray et al, 2022; Rhim et al, 2014). While aligned collagen architectures promote carcinoma invasion (Ray et al, 2022; Ray et al, 2017), the impact of the extensive PDA collagen network on T cell movement for tumor sampling remains elusive. Utilizing the T cell dynamics assay (Fig. 1) with CD8 + T cells, we first define the spatial distribution of T cell movement relative to the collagen network (Fig. 2A; Movie EV1). We observed heterogeneous T cell behaviors, with two predominant behaviors: regions of T cell trapping, characterized by highly restricted movement within confined zones, and contact guidance, marked by T cell movement along defined structural cues. In this latter context, contact guidance-directed migration results in T cells moving along fiber networks throughout the stroma and being guided past carcinoma cells (e.g., Fig. 2A, path 1), but in other cases can direct T cells toward carcinoma cells (e.g., Fig. 2A, path 2). Hence, collagen architecture may have both negative and positive consequences. To explore this further, we next sought to quantitatively define the interactions between T cells and collagen fibers and the influence of ECM architecture on directed migration.

Temporal quantification of T cell infiltration demonstrates that CD8 + T cells are predominantly associated with nearby collagen signals at all time points, indicating that T cell migration in the PDA TME strongly favors collagen (Fig. 2B). Indeed, even in regions rich in carcinoma cells, T cells primarily remain localized within collagen-rich subregions, while carcinoma dense regions that lack prominent collagen signals are largely devoid of T cells (Fig. 2C). Notably, for non-trapped T cells, aligned collagen fibers promote directional and ballistic T cell migration that influences the localization and tumor area sampling by T cells. Thus, the collagen network effectively functions as a high-affinity microscopic highway network within the stroma that can facilitate rapid effector T cell movement but also limit their ability to deviate from structured paths to search for cognate antigens. To capture this behavior, we define the tendency of a T cell failing to deviate from a set path due to the underlying collagen fiber directionality as migration anisotropy. To metricize migration anisotropy, we first tracked T cell migration on 2.5D nano-patterned substrates that mimic tumor collagen fiber spacing to provide a substrate with a defined anisotropy axis (Tabdanov et al, 2021) (Fig. 2D). Quantitative analysis of T cell tracks shows that T cell motility on anisotropic substrates is indeed directionally biased (Fig. 2D,E). Fundamentally, the physical aspects of migration anisotropy dictate that deviations from the ECM alignment axis are physically unfavorable, creating mechanoresistance (mR) for off-axis traversal. To further elucidate the effect of mR and its spatial distribution around carcinoma cells, we developed the MechanoTrack analysis algorithm and computationally mapped mechanoconductance (mC), the mathematical inverse counterpart of mR, for PDA tumors (Fig. 2F,G). By integrating T cell tracks from polyclonal wild-type (WT) CD8 + T cells in PDA tumors, MechanoTrack records step-wise mC terrain information for each timestamp, providing an objective measure of ECM-facilitated “immune distraction” based on the principle of migration anisotropy. Therefore, we hypothesized that the ECM architecture can dictate the accessibility of infiltrating T cells solely based on its position relative to carcinoma cells, creating spatial heterogeneity for potential infiltration routes and consequently “physical immunosuppression” (Fig. 2H).

Quantitative analysis of the CD8 + T cell migration time series data using MechanoTrack reveals that collagen architectures often lead to movement characterized by ECM-guided meandering behavior or moving past or around target carcinoma cells (e.g., Fig. 2A, path 1) rather than productive sampling of carcinoma targets. This is largely due to the propensity of T cell movement along high-mC regions associated with collagen fibers (Fig. 2I). By overlaying T cell migration tracks with topological mC plots, we found that T cells that are more migratory and mostly travel along the topological ridges (high mC) instead of toward topological valleys (i.e., lateral off fiber movement toward low mC regions; Fig. 2I). We further examined the tendency of T cells to overcome mR barriers and sample both high-mC and low-mC regions (i.e., cross-sampling), as compared to remaining in either region alone (i.e., mono-sampling). We discovered that T cells predominantly engaged in mono-sampling rather than cross-sampling (Fig. 2J,K), suggesting that once a T cell is engaged in the collagen fiber network, it is difficult to dissociate from that interaction and effectively sample adjacent carcinoma regions. Collectively, these findings illustrate the biophysical challenges that infiltrating T cells often face in solid tumors with desmoplasia, such as PDA. They signify that infiltrating T cells in the PDA are biased to follow the direction of ECM fibers, which renders T cell sampling and coverage highly inefficient, creating accessibility cold spots for targeting carcinoma cells (i.e., immune exclusion zones). Overall, these findings highlight a nuanced and often counterintuitive role of collagen in T cell movement within PDA. While collagen fibers can act as high-affinity highways guiding T cells through the stroma, they also impose significant off-axis resistance, thereby selectively constraining T cell sampling of the tumor volume and therefore limiting engagement with carcinoma cells.

### Collagen and immunosuppressive myeloid cells form coordinated obstacles that dampen engineered T cell sampling of PDA

**Figure 3:**
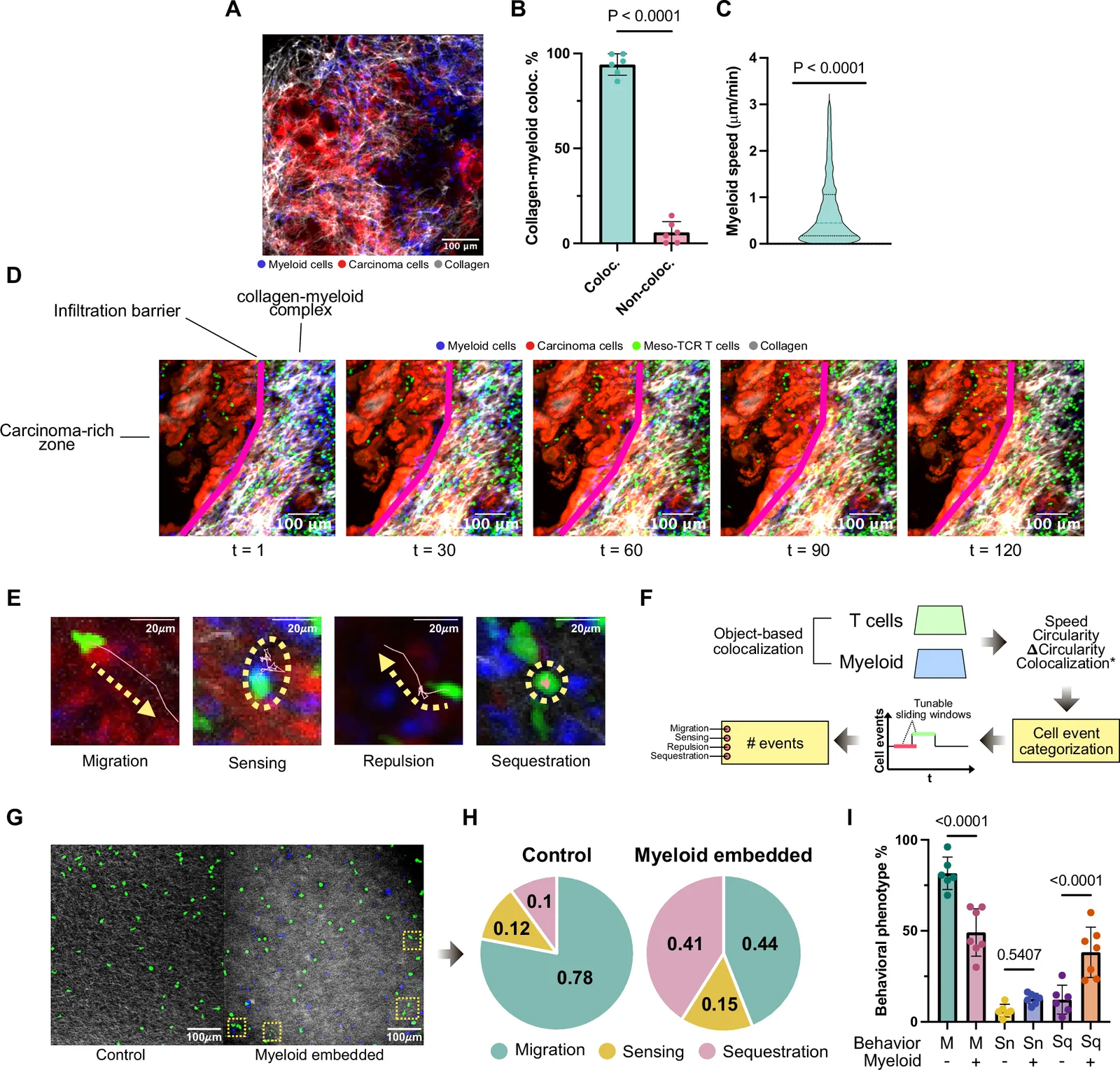
Collagen and myeloid cells form coordinated immunosuppressive obstacles. (**A**) MPLSM of live-tumor with labeled CD11b+ myeloid cells (blue), carcinoma cells (red), and collagen fibers (gray) shows the abundance of immunosuppressive myeloid cells in the TME. (**B**) Myeloid cells and collagen are highly colocalized (coloc.=94.2%, *P* < 1e-15, *n* = 6, unpaired *t* test). (**C**) Mean track displacement distances of myeloid cells (μ = 2.373 μm, unpaired *t* test) show non-zero (*P* = 2.1e-1472, *n* = 11227, one-sample *t* test) but slow migration speed (μ = 0.7148 μm/min). (**D**) Meso-TCR CD8 + T cells 4D series (2-hr) in *KPCT* tumor slices (green = meso-TCR T cells; blue = CD11b+ myeloid cells; red = carcinoma cells; gray = collagen). The yellow line shows a clear demarcation between the carcinoma-rich zone and the collagen-myeloid complex due to collagen fibers. (**E**) Frequently observed T cell behaviors (migration, sensing, repulsion, and sequestration) show heterogeneity in T cell–ECM interaction (pink = tracks, yellow arrows = movement, yellow circles =  boundaries). (**F**) Schematics of PhenoTrack. Cell events are categorized and extracted using a convolution operation with a tunable window using cell velocity, circularity, change in circularity, and colocalization from time series. (**G**) Meso-TCR T cell migration in tumor-derived myeloid-embedded collagen matrix (MECM), showing instances of T cell fragmentation (yellow boxes). (**H**) Case study shows increased sequestration (control = 10%/MECM = 41%) and decreased migration (control = 78%/MECM = 44%). (**I**) Percentage migration, sensing, and sequestration events across all samples support the finding in the case study with increased sequestration (*n* = 7, *P* = 1.9e-6, one-way ANOVA with multiple comparisons) and decreased migration events (*n* = 7, *P* = 7.2e-5, one-way ANOVA with multiple comparisons). Error bars represent standard deviation. Source data are available online for this figure.

While collagen architecture significantly restricts cytotoxic T cell sampling, there is abundant evidence that collagen alone does not entirely explain the robust immunosuppression observed in the PDA (Beatty et al, 2015; Dixit et al, 2022; Panni et al, 2019; Stromnes et al, 2014). Myeloid cells, including TAMs and MDSCs, are CD11b-positive cells that are abundantly present in the PDA stroma (TAMs and MDSCs make up >95% of CD11b+ cells in PDA (Dixit et al, 2022; Elyada et al, 2019)), and their presence is associated with profound immunosuppression (Beatty et al, 2015; Dixit et al, 2022; Panni et al, 2019; Stromnes et al, 2014). The percentage of classically activated TAMs is extremely low (Dixit et al, 2022; Elahi-Gedwillo et al, 2019; Elyada et al, 2019). However, the interplay between T cell and myeloid cell dynamics with respect to ECM architecture in space and time remains unclear. We hypothesized that one key tactic employed by immunosuppressive myeloid cells in the stroma is to alter therapeutic T cell behavior and inherently dampen T cell anti-tumor immunity by interfering with T cell search strategies. To define this, we evaluated the coordinated dynamics of these three key elements. Consistent with previous reports (Beatty et al, 2015; Dixit et al, 2022; Panni et al, 2019; Stromnes et al, 2014), we observed a robust presence of myeloid cells. Immunofluorescence staining of live tumor slices from *KPCT* mice showed that CD11b+ myeloid cells are ubiquitous in the PDA TME (Fig. 3A). Object-based colocalization of CD11b and collagen fibers found that CD11b+ myeloid cells colocalize with collagen fibers at a surprisingly high frequency (>94.2%; Fig. 3B). In addition, relative to the speeds of migrating T cells in PDA (Tabdanov et al, 2021), myeloid cell migration is extremely limited (myeloid cells are ~10–20× slower than migrating T cells). Together, this suggests that myeloid cells could function as roadblocks that physically and chemically interact with cytotoxic T cells traveling along collagen networks and, therefore, obstruct T cell movement and target-driven infiltration into carcinoma zones. To test this hypothesis, in live PDA, we examined the movement of therapeutic CD8 + T cells engineered to express a high-affinity Msln_406-414_:H2D^b^-specific TCR (clone 1045) against murine mesothelin (i.e., meso-TCR T cells), an established tumor-associated antigen in PDA (Stromnes et al, 2015). Consistent with our data showing T cells localizing predominantly in collagen-rich stromal regions (Fig. 2) and colocalization of TAMs/MDSCs with collagen fibers (Fig. 3A–C), T cells in *KPCT* tumors remain predominantly confined within the collagen-myeloid-rich regions and rarely disperse throughout the tumor volume over time (Fig. 3D; Movie EV2). These findings suggest an additional major barrier to T cell sampling of carcinoma regions, where collagen and immunosuppressive cells are co-conspirators to form a dual obstacle that restricts effective T cell movement.

To explore this complex immunosuppressive behavior in more detail, we further explored single-cell spatiotemporal dynamics. We observed that at the single-cell level, T cell behaviors can be generally categorized into four distinct behaviors (Fig. 3E): (1) migration characterized by rapid movement as T cells traverse the environment; (2) sensing defined by limited overall cell displacement coupled with prominent protrusions reflected by continuous changes in circularity to probe their more immediate surroundings; (3) repulsion indicated by a change in the direction of T cell movement after a T cell-myeloid colocalization, and (4) sequestration marked by a cessation of movement and rounded morphology. It is important to note that T cell behaviors and interactions with the TME do not happen instantaneously, underscoring the need for temporally resolved analysis. Thus, to capture these complex temporal behaviors, we developed PhenoTrack, an algorithm that categorizes and tracks cell behaviors with target awareness through object-based colocalization (Fig. 3F). Using TrackMate-Weka for machine learning (ML)-based T cell segmentation, we tracked T cell coordinates and morphology over time, including frame-to-frame changes in circularity to capture protrusive activity to categorize temporal T cell behaviors into quantitated events. To test this analysis pipeline and confirm that myeloid immunosuppression and ECM-based immunosuppression are, in fact, additive, we first adopted a reductionist approach by isolating CD11b+ myeloid cells directly from *KPC* tumors (i.e., TAMS and MDSCs) and embedding them within 3D collagen matrices (Fig. 3G). Multiphoton imaging of meso-TCR T cells in myeloid-embedded collagen matrix (vs. Control) followed by PhenoTrack analysis reveals spatially and temporally distinct behaviors (Fig. 3H,I). While there is no significant difference in T cell sensing, we defined a significant decrease in T cell migration and a profound increase in T cell sequestration in both a representative case study (Fig. 3G,H) and across all groups (Fig. 3I), suggesting that immunosuppressive myeloid cells in the PDA TME not only chemically impair T cell migration but also can directly halt effective T cell movement by promoting sequestration.

### ECM architecture leads to increased T cell-myeloid cell contact and immunosuppression

**Figure 4:**
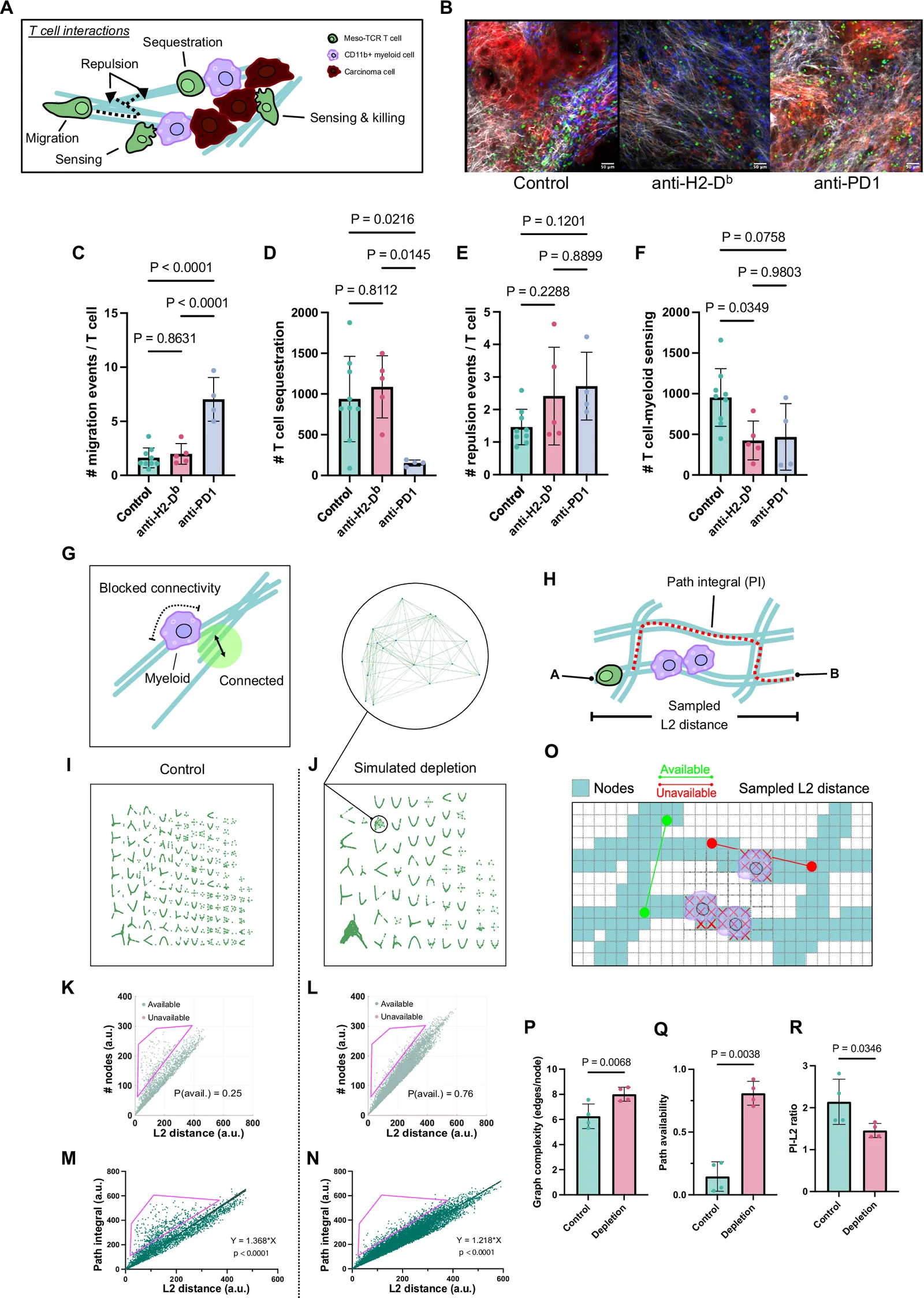
ECM architecture influences carcinoma-immune spatiotemporal relationships and interactions. (**A**) Predominant T cell interactions observed in the T cell dynamics assay. (**B**) MPLSM of T cell dynamics assays under Control, MHC-I (H2-D^b^) blockade, and ICB (PD1). (**C**–**F**) MHC-I blockade with H2-D^b^ neutralizing antibody decreases T cell-myeloid cell sensing events (*P* = 0.0349, one-way ANOVA with multiple comparisons). ICB significantly increases migration events (*n* = 4, *P* = 5.7e-5, one-way ANOVA with multiple comparisons) and decreases T- cell sequestration (*n* = 4, *P* = 0.0216, one-way ANOVA with multiple comparisons). (**G**) Myeloid cells on collagen fibers block essential paths, resulting in forced tortuosity and increased myeloid suppression. (**H**) Disconnected ECM paths due to myeloid blockades impose forced tortuosity on T cells, which increases the distance traveled (PI) compared to the straight line distance (L2 distance) and hence the PI-L2 distance ratio. (**I**, **J**) Undirected graphs, shown in green clusters, constructed from collagen fibers in PDA tumors, show increased levels of path fragmentation in (**I**) the control group, compared to (**J**), simulated depletion of myeloid cells. (**K**, **L**) BFS algorithm and Monte Carlo simulation quantitatively show the number of nodes (*y* axis) required for T cells to travel a randomly sampled L2 distance (*x* axis). Simulated depletion of myeloid cells enhances fiber connectivity and availability (76%) compared to the control group (25%). Upward scattering of sampled points (pink boxes) demonstrates that myeloid cells decrease the efficiency of T cell routing without myeloid depletion. (**M**, **N**) Using the BFS algorithm on the graphs combined with Monte Carlo simulation shows similar upward scattering of sampled instances (pink boxes), quantitatively confirming that T cells require a longer path to cover the sampled L2 distance with myeloid cells present (*P* < 1e-15, F-test) compared to simulated depletion (*P* < 1e-15, F-test). (**O**) Path availability and forced tortuosity. (**P**, **Q**) Simulation indicates that removing myeloid cells increases graph complexity (*n* = 4, *P* = 0.0068, unpaired *t* test) and path availability (*n* = 4, *P* = 0.0038, unpaired *t* test) by making the fiber network more connected. (**R**) Simulated depletion of myeloid cells decreases the path integral to L2 distance ratio (*n* = 4, *P* = 0.0346, unpaired *t* test). A higher PI-L2 ratio indicates less efficiency due to the increased length needed to travel a unit distance. Error bars represent standard deviation. Source data are available online for this figure.

To further define T cell–ECM-myeloid interactions using our platform, we analyzed live tumors using meso-TCR T cells (Fig. 4A) and quantified T cell dynamics following pharmacologic perturbations to influence critical mechanisms by which CD11b+ myeloid cells may mediate immunosuppression (Fig. 4B). Namely, we hypothesized that T cell-myeloid cell interactions are partly driven by MHC-I-dependent antigen presentation or immune checkpoint interactions, where myeloid cells in PDA are known to express high levels of PD-L1 (Stromnes et al, 2017). Quantitative characterization of T cell migration using PhenoTrack demonstrates that MHC-I restriction using a blocking antibody to H-2D^b^ (B22-249.R1) does not significantly alter the number of migrating T cells nor myeloid sequestration, while myeloid repulsion events show a moderate trend (Fig. 4C–F). However, myeloid sensing notably decreases (Fig. 4F), consistent with a decline in cell–cell interactions. In comparison, ICB drastically increases the number of migrating T cells and alleviates myeloid sequestration, while showing a moderate trend for increased repulsion and a stronger trend for decreases in sensing (Fig. 4C–F). Together, these data indicate that, in addition to established chemical immunosuppressive signals (e.g. (House et al, 2020; Ray et al, 2024)), direct contact with myeloid cells can also influence T cell behaviors. Indeed, the significant decrease in myeloid sequestration under ICB underscores that myeloid cells can have potent effects on T cell sampling by rapidly shutting down movement based on physical contact.

Thus, to examine the multi-prong immunosuppression by myeloid cells in the PDA TME and quantitatively investigate the extent of suppression facilitated by the interconnected myeloid-populated collagen highways, we hypothesized that the strong spatial colocalization of immunosuppressive myeloid cells and collagen fibers could reduce the availability of migration paths that can be traveled without encountering myeloid interference. This is indicated by a reduction in path availability between two selected points A and B, thereby forcing T cells to halt, reverse direction, or take suboptimal detours during myeloid encounters (Fig. 4G). Indeed, myeloid presence inflicts forced tortuosity, defined here as the extent of detour that an infiltrating T cell must take to navigate between two points instead of directly traveling the shortest L2 distance, or mathematically, the ratio between the path integral and its L2 distance (Fig. 4H). This forced tortuosity negatively affects T cell search strategies by obstructing T cell migration through physically and chemically myeloid roadblock points. To quantitatively determine such impact from the lens of the infiltrating T cells, we employed computational simulations that are directly informed by collagen and myeloid coordinates derived from imaging data of *KPCT* tumors (Fig. 4I–R). We assessed how both the presence and simulated depletion of immunosuppressive myeloid cells influence path availability and forced tortuosity. Undirected graphs were constructed using adjacency matrices in which each segmented collagen pixel accounted for a node (Fig. 4O). Internodal distances in the graph served as weights, emphasizing closer connections and penalizing distant connections to represent node-to-node strength. As a result, we found that myeloid-laden collagen networks (i.e., Control conditions) have fewer connected edges per node (Fig. 4I,P), indicating that these networks are more fragmented compared to simulated full depletion of myeloid cells (Fig. 4J,P). Next, using Monte Carlo simulation, we numerically assessed the path availability and forced tortuosity by randomly sampling 10,000 coordinate pairs from the tumor-derived weighted graphs and finding the shortest path by breadth-first search (BFS). L2 distance sweep shows decreased percentage of available paths (Fig. 4K,L,Q) for control vs. myeloid depletion. Additionally, sampled points in the control group show a notable propensity for upward scattering (Fig. 4K), compared to simulated depletion of myeloid cells (Fig. 4L), indicating that T cells are compelled to utilize more nodes to cover the same start-to-endpoint distance. Using the same principle, for each sampled coordinate pair, we calculated the forced tortuosity by finding the ratio of the path integral (PI) and its L2 distance (Fig. 4M,N,R). Consistent with path availability, upward scattering of sampled instances was observed in the control group (Fig. 4M,R), indicating that T cells are forced to traverse a longer path in the control group to cover the same L2 distance, compared to simulated myeloid depletion (Fig. 4N,R). Overall, the numerical simulation quantitatively shows that the presence and localization of myeloid cells decrease available paths for efficient T cell travel through the collagen matrix, which further supports the rationale for TAM and MDSC depletion in PDA.

### Deep learning identifies TME features that regulate T cell dynamics and suppression

**Figure 5:**
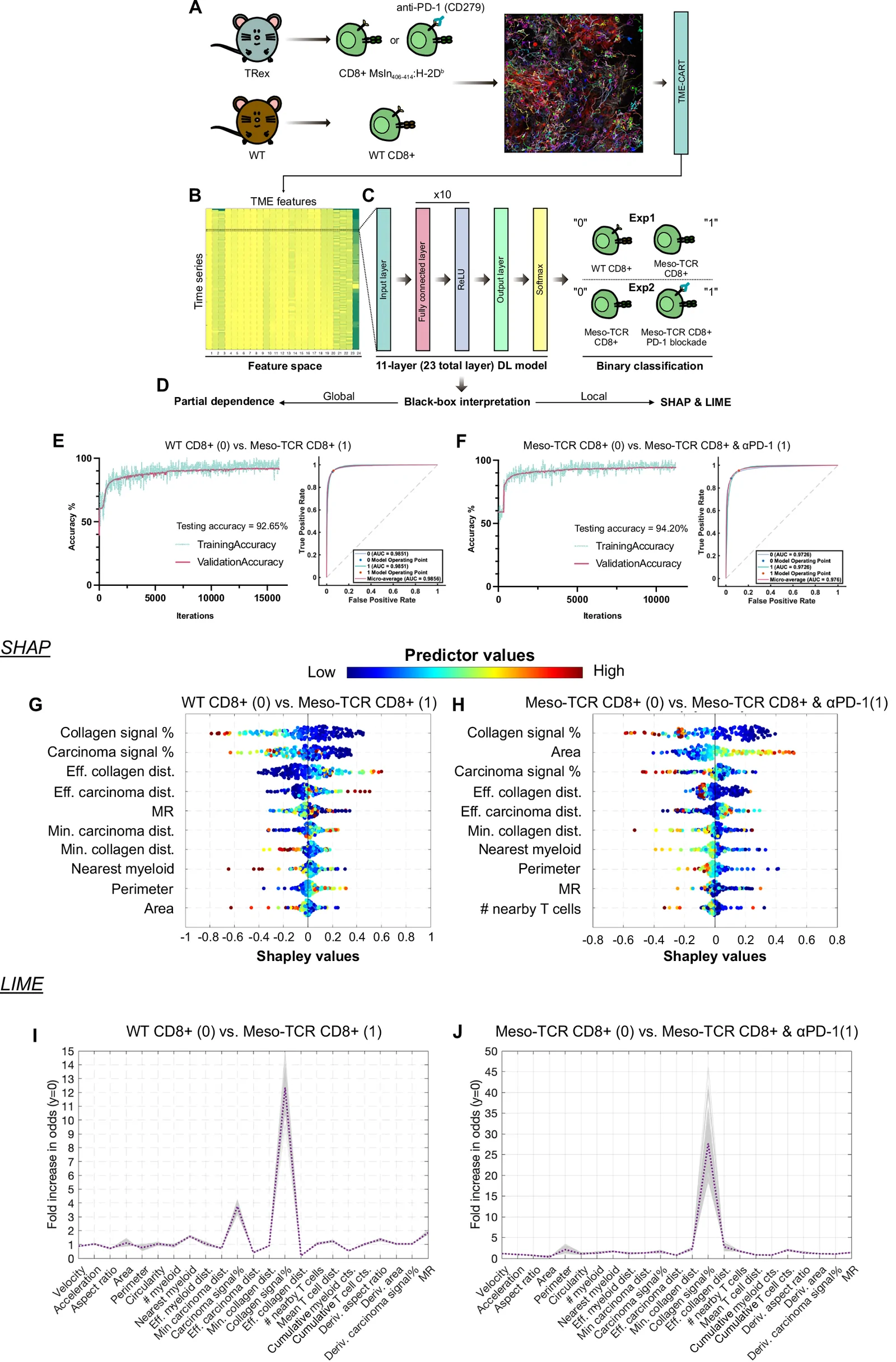
Deep learning to identify key features that regulate T cell behavior. (**A**) Imaging data from T cell dynamics assays (i.e., WT and meso-TCR CD8 + T cells from TRex mice and meso-TCR + /- ICB) are processed with the TME-CART pipeline. (**B**) A 23-dimensional feature space constructed from TME-CART (Table EV1). (**C**) A fully connected neural network architecture for binary classification is applied on experiment datasets. (**D**) DL models are explained by SHAP, LIME, and PDPs for local and global interpretations of T cell-TME interactions. (**E**, **F**) Accuracy plots for both models during the training and validation process, showing convergence of the model with testing accuracy at 92.65% and 94.2% for WT CD8 + /meso-TCR CD8+ and meso-TCR CD8 + / meso-TCR & PD-1 blockade, respectively. AUC-ROCs show excellent classification performance for both DL models. (**G**, **H**) SHAP (*n* = 200) on both datasets quantitatively reveals and ranks the top ten contributing factors for changes in T cell-TME interactions driven by antigen recognition and immune checkpoint. (**I**, **J**) Aggregate LIME coefficient plots (*n* = 100) show mean fold increase in odds (purple dashed line) for each feature that would sway the model’s prediction towards *y* = 0 (i.e., WT CD8+ or meso-TCR CD8+ w/o ICB). Source data are available online for this figure.

To decipher how the immunosuppressive TME components and T cell dynamics spatially and temporally influence T cell tumor sampling, we further analyzed live tumor imaging data for WT and meso-TCR CD8 + T cells, as well as meso-TCR T cells with and without ICB (Fig. 5A). We compiled and feature-engineered each dataset into a high-dimensional feature space from TME components, as well as derivatives and integrals of selected features to account for temporal dependencies (Fig. 5B and Table EV1). We trained an 11-layer (23 total layers) deep neural network for each experimental condition (Fig. 5C and Table EV2), followed by SHapley Additive exPlanations (SHAP) and Local Interpretable Model-agnostic Explanations (LIME) for black-box explanations, as well as Partial Dependence Plots (PDPs) for identifying trends at a global output level (Fig. 5D), in order to distill key cellular and stromal factors from deep learning (DL) models (Friedman, 2001; Lundberg and Lee, 2017; Ribeiro et al, 2016). Training of the DL models using the prepared datasets shows high testing accuracy (92.65% and 94.20%), which is also reflected by AUC-ROC values of 0.9856 and 0.9760, respectively (Fig. 5E,F). Training–validation–testing split before training and early stopping during training are used to enhance the generalizability of the DL model.

**Figure EV1:**
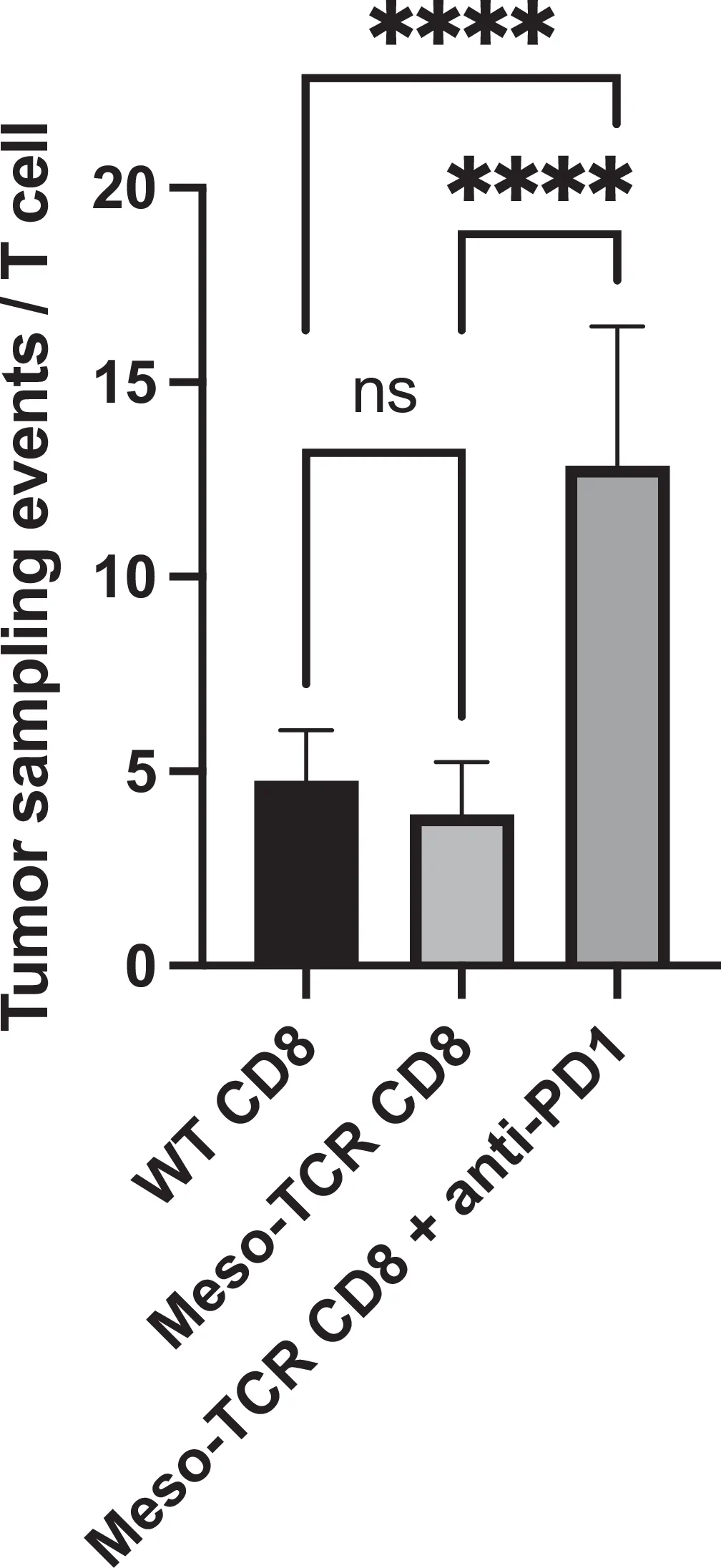
Anti-PD-1 increases meso-TCR CD8 + T cells tumor sampling compared to WT CD8 + T cells (*n* = 7, ****P < 0.0001, one-way ANOVA w/multiple comparison) and meso-TCR CD8 + T cells alone (*n* = 6, ****P < 0.0001, one-way ANOVA w/multiple comparison). Error bars represent standard deviation.

Post hoc interpretation using SHAP on 200 randomly sampled instances in each dataset shows the top ten stromal and cellular factors contributing to model prediction (Fig. 5G,H). Based on game theory, a positive Shapley value in a binary classification model indicates that the feature pushes the prediction toward the positive classes (i.e., meso-TCR CD8+ or less suppressed T cell due to ICB), while a negative Shapley value indicates the opposite (e.g., low collagen signal % values correspond to positive Shapley value, indicating that meso-TCR CD8 + T cells are more often associated with lower collagen signal). Thus, based on such interpretations, SHAP results indicate that meso-TCR T cells are strongly associated with a lower percentage of collagen signal in their immediate vicinity, suggesting that they are more effective at traversing off the dense collagen network than WT T cells. Furthermore, meso-TCR T cells with ICB exhibit an even greater distance from collagen-rich regions (Fig. 5H), suggesting that ICB could also help reduce stromal trapping and thus enhance T cell movement through challenging environments. This trend persists for the minimum collagen distance in both groups, which suggests that meso-TCR T cells can more frequently reside in collagen-sparse neighborhoods, and ICB further enhances such effect. Likewise, examination of carcinoma signal percentage, which represents the proportion of local carcinoma signal at each time point, suggests that meso-TCR T cells are more often found in local neighborhoods with lower carcinoma signal % (Fig. 5G,H). This is consistent with increased volume sampling from less constraint from the collagen matrix and increased ability to localize near sparsely positioned carcinoma cells. However, immunosuppression from the collagen network and myeloid cells remains, but can be reduced through ICB (Fig. EV1). Myeloid cells are more distant from T cells with ICB (Fig. 5H), consistent with less sequestration and more T cell movement. Taken together, feature extraction from SHAP successfully captures shifts in T cell behavior in PDA.

**Figure EV2:**
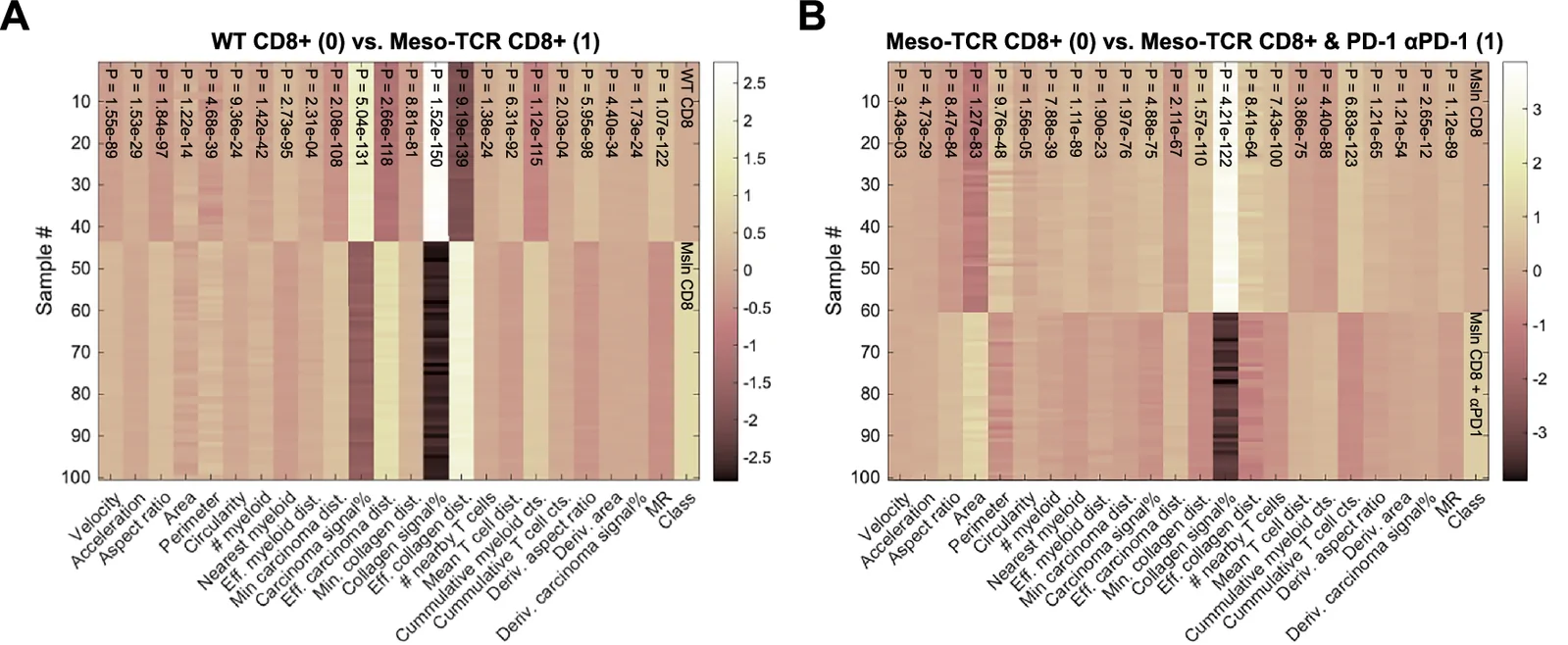
LIME coefficient analysis. (**A**, **B**) Aggregate LIME plots of 100 randomly sampled points (WT CD8+ | Meso-TCR CD8 + ) and (Meso-TCR CD8+ | Meso-TCR CD8+ with ICB) groups, sorted by class. *P* values show the extent of the difference in mean LIME coefficients between classes.

To further quantitatively evaluate the impact of each feature on the prediction of the DL models and corroborate SHAP findings, we calculated LIME coefficients for 100 randomly sampled instances (Figs. 5I,J and EV2A,B). LIME feeds the model with perturbations in the input and then monitors how the model’s predictions vary with these perturbations. It subsequently fits an interpretable model (e.g., linear regression) to approximate the black-box model’s behavior within that small neighborhood of feature space. For instance, the percentage collagen signal in the local neighborhoods increases the likelihood of a y=0 model prediction (i.e., suppressed behavior) by 12.3- and 27.6-fold. Overall, the LIME analysis largely mirrors interpretations concluded using SHAP. The spatial relationships of collagen, carcinoma cells, myeloid cells, and mR all emerge as prominent factors, with decreased mR associated with meso-TCR cells vs. WT, again highlighting that while the stromal barriers remain very limiting for T cells under all conditions, meso-TCR cells are less constrained. The significance of these findings is multifold for interpreting local T cell dynamics. First, collagen emerges as the most influential factor in both analyses. It shows that the collagen fiber network not only forms a physical barrier but also harbors key immunosuppressive myeloid cells, and may structurally promote T cell aggregation, all of which substantially modulate T cell behavior. Second, it is shown that enhanced T cell activation driven by the presence of cognate antigen and ICB helps reduce suppression, thereby increasing the potential for T cell anti-tumor responses. Finally, enhanced T cell activation by the presence of antigen and ICB can reduce collagen-induced migration anisotropy that often restricts T cell movement. Therefore, the analysis pipeline predicts that therapeutic strategies that selectively perturb collagen architecture and immunosuppressive myeloid cells, combined with focused T cell engineering strategies, have strong potential to enhance T cell penetration and tumor volume sampling.

### One- and two-variable partial dependence plots (PDPs) reveal nonlinear synergistic ECM–cell interactions shaping T cell behavior

**Figure 6:**
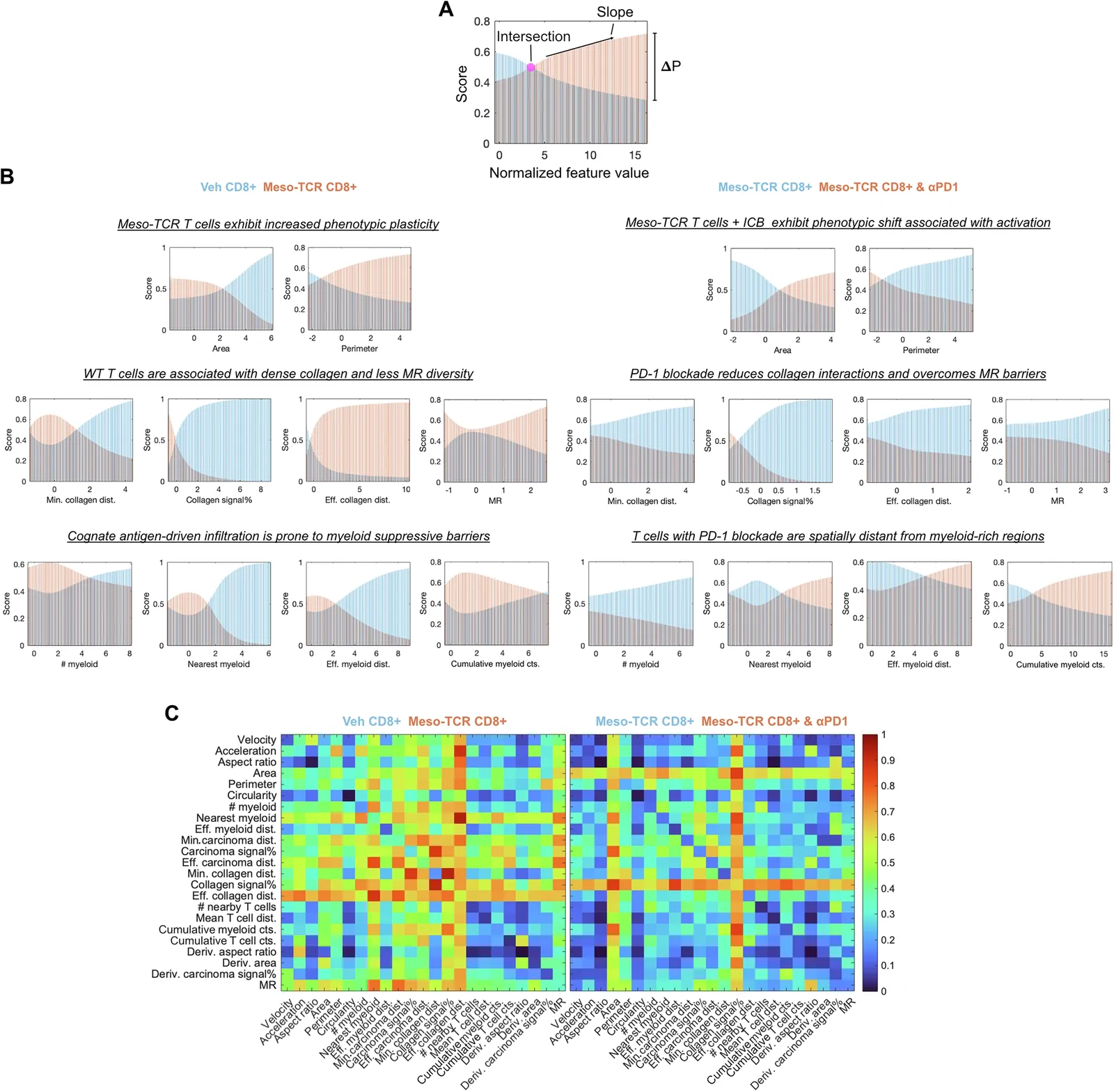
Partial dependence plots reveal nonlinear factors influencing T cell behavior. (**A**) One-variable PDPs show how the probability of a prediction changes as a feature increases or decreases numerically at the model output level. Intersection, slope, and difference in P (difference in PDP scores) of PDPs are indicators of T cell-TME interactions. (**B**) Selected one-variable PDPs for meso-TCR CD8 + /meso-TCR CD8+ and meso-TCR CD8 + /meso-TCR CD8+ & ICB experiments (see also Fig. EV3A,B). (**C**) Two-variable PDP heatmaps show maximum differences in probability of predictions towards either class due to the combinatorial effect of two variables. A red shift (1) indicates a larger impact on the model’s prediction due to the combination, while a blue shift (0) indicates a smaller impact. Source data are available online for this figure.

**Figure EV3:**
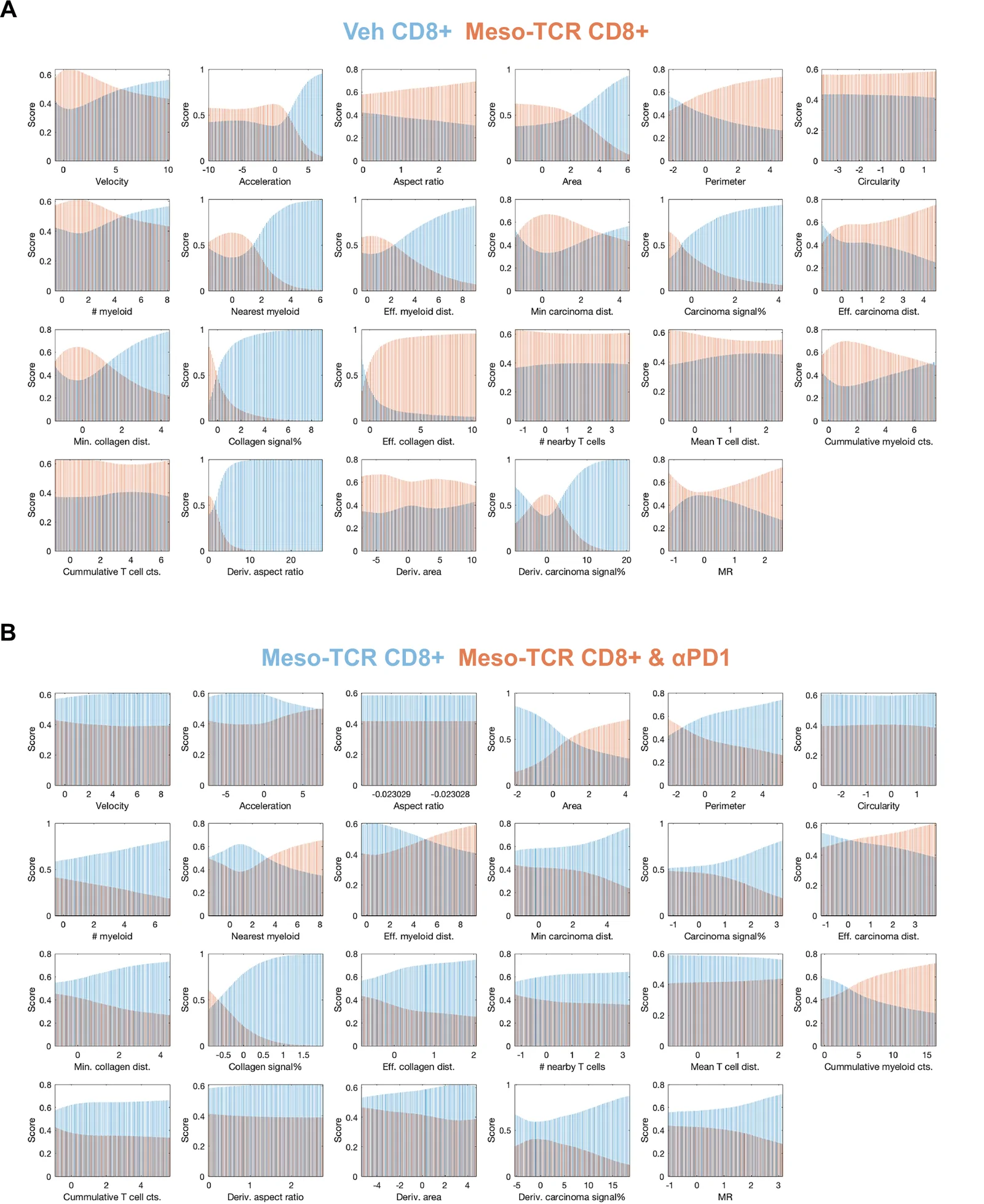
Partial dependence plots. (**A**, **B**) Full PDP panels with 23 features included in the DL training (WT CD8+ | Meso-TCR CD8 + ) and (Meso-TCR CD8+ | Meso-TCR CD8+ with ICB) experiments.

To investigate global trends in T cell dynamics in relation to TMEs, we employed PDPs with our DL models (Figs. 6A,B and EV3). In contrast to local interpretations, which focus on explanations localized to an individual point, PDPs can uncover nonlinear interactions at the global model-output level. In PDPs, the *x* axis represents normalized feature values, while the *y* axis score reflects the predicted probability for a specific class (Fig. 6A). Key interpretative elements in a PDP include the point of intersection, which marks the threshold at which the model shifts its prediction from favoring one class to another, and the rate of change, or slope, which reflects the sensitivity of the model’s prediction to changes in the feature value, as a steeper slope indicates that even a small change in the feature can significantly affect the predicted outcome. Conversely, the difference in probability allows us to peek into the trend as the feature value varies, highlighting the magnitude of its impact and the specific points where that impact occurs numerically. Nonlinearities such as plateaus, valleys, and curvatures may further signal complex relationships and interactions (Fig. EV1).

Comparing the morphological features of WT with meso-TCR T cells, PDP scores (Fig. 6B) show that an increased aspect ratio, perimeter, and frame-to-frame changes in cell area are associated with meso-TCR T cells, suggesting that these cells are more phenotypically plastic, a feature that promotes movement in complex environments (Tabdanov et al, 2021), even though their overall cell area was reduced compared to WT cells. Regarding T cell-collagen interactions, WT T cells were highly associated with an increased percentage of collagen signal, consistent with local interpretations (Fig. 5), evident from the monotonic increase and rapid saturation of its probability curve, and consistent with local interpretations (Fig. 5G–J). This again indicates that WT T cells were more likely to be associated with dense ECM. Moreover, the nuanced and perhaps non-intuitive nature of T cell-stromal interactions is also reflected in the PDP for mR, showing a biphasic trend in which meso-TCR T cells are more associated with collagen-adjacent regions that possess either high or low mR. Notably, the myeloid PDPs suggest that meso-TCR T cells are distinguished less by residence within the myeloid-rich regions and more by proximity to nearby myeloid cells. This pattern is in line with our earlier observations that CD11b+ myeloid cells are highly colocalized with collagen fibers and function as approximately immobile, suppressive roadblocks within the ECM. Thus, it suggests that when cognate antigen-driven meso-TCR T cells actively probe the stroma–carcinoma interface, these T cells appear more likely to be intercepted by strategically positioned myeloid cells, promoting inefficient behavioral phenotypes such as sensing and sequestration, rather than tumor sampling. Comparing meso-TCR T cells with and without ICB (Fig. 6B), we found that T cells under checkpoint blockade exhibit increased influence of area despite a reduction in perimeter, again suggesting a shifting phenotype. Consistent with SHAP and LIME, PDPs suggest that ICB reduces collagen interactions, which is further supported by PDP outputs showing that meso-TCR T cells without ICB are associated with high mR. Regarding myeloid suppression, the addition of ICB displaced T cells further from myeloid cells, suggesting they can successfully disengage myeloid suppression, reducing the likelihood of sequestration within myeloid-rich local neighborhoods, despite a higher frequency of myeloid cell encounters overall.

To gain deeper insight into complex multivariate interactions, we analyzed how features synergistically interact and influence T cell dynamics. We calculated two-variable PDPs to visualize the combined effect of two selected feature pairs on the model predictions (Fig. 6C). The resulting heatmap, which can also be used to identify global pattern shifts to evaluate the impact of different conditions, illustrates the maximum change in prediction probability, expressed as $\max (\Delta P)$. A red shift indicates a greater impact of the specific two-variable combination on the model’s prediction (i.e., immunosuppression), while a blue shift denotes a smaller effect. In comparing WT and meso-TCR T cells, effective collagen distance pairing with all other features consistently influences the model’s prediction to a great extent. The most substantial shift in prediction occurred when effective collagen distance was combined with nearest myeloid cells and acceleration, suggesting that, consistent with our finding of rapid movement along collagen fibers, the ability to exert force in collagen-myeloid complexes is a defining factor, which is also validated by mR-collagen pairs. The combination of effective collagen distance with morphology metrics also led to a notable shift, further underscoring the complex role of collagen in either facilitating or hindering T cell infiltration. Feature connectivity between effective collagen distance and the nearest myeloid distance or minimum carcinoma distance again emerged as evidence that antigen-activated T cells behave differently when interacting with carcinoma-ECM and myeloid-ECM complexes. Likewise, for meso-TCR T cells with and without ICB, both area and percentage collagen signal emerge as powerful features, forming pairs across all features and influencing the model’s predictions. In terms of area pairings, we found that nearest myeloid distance, percentage carcinoma signal, percentage collagen signal, and cumulative myeloid cell encounters are the most significant co-features distinguishing immune checkpoint dynamics. Thus, the intricate interplay among carcinoma cells, CD11b+ myeloid cells, and the surrounding ECM architecture is illustrated by the prominently red-shifted clusters. Together, the evidence suggests that ECM architecture is not only immunosuppressive on its own but also collaborates with carcinoma cells, myeloid cells, and collagen to form a multifaceted barrier that affects antigen-activated T cell behavior, revealing that antigen-activated T cells can invoke high-order behavioral shifts that are not easily accessible through conventional statistics.

### TME-CART deep learning captures the impact of myeloid depletion on decreased T cell suppression and enhanced tumor sampling

**Figure 7:**
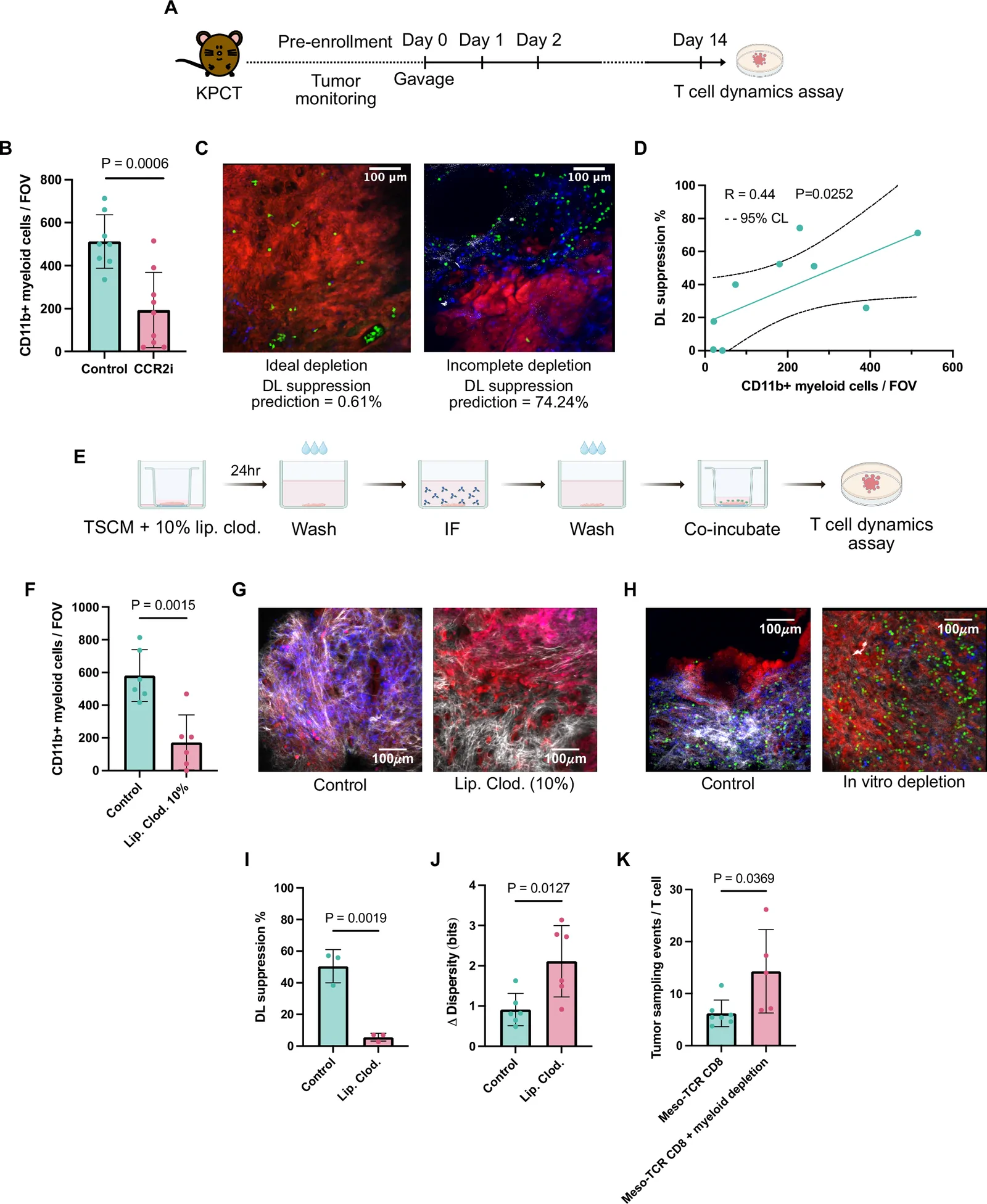
The TME-CART deep learning framework accurately predicts shifts in immunosuppression following depletion of myeloid cells. (**A**) In vivo myeloid depletion in *KPCT* mice using CCX-598 followed by T cell dynamics assays. (**B**) Quantifying the number of myeloid cells shows a significant but heterogeneous reduction in myeloid cells (*n* = 9, *P* = 0.0006, unpaired *t* test). (**C**) MPLSM of T cell dynamics assays in CCR2i-treated mice show regions of robust and incomplete depletion with associated levels of immune suppression predicted by the DL model. (**D**) Suppression predicted by the DL model with regard to the number of myeloid cells reveals a moderate correlation (*n* = 8, *R* = 0.44, “center”: linear regression, “--”: 95% confidence level, *P* = 0.0252 with Spearman Correlation). (**E**) Ex vivo depletion of myeloid cells in PDA tumor slices using 10% w/v liposomal clodronate, followed by myeloid labeling and MPLSM. (**F**) Liposomal clodronate depletion significantly reduces myeloid cells and results in less heterogeneity compared to CCR2i (*n* = 6, *P* = 0.0015, unpaired *t* test). (**G**) MPLSM of myeloid-depleted tumor slices using 10% w/v liposomal clodronate compared to control tumor slices. (**H**) T cell dynamics assay using meso-TCR T cells on control and ex vivo myeloid-depleted tumor slices. (**I**) Depletion of myeloid cells significantly reduces immune suppression predicted by the DL model (*n* = 3, *P* = 0.0019, unpaired *t* test). (**J**) Using K-L k-NN entropy estimator, changes in T cell dispersity indicate better T cell dispersal in the myeloid-depleted tumor slices than in control tumor slices (*n* = 6, *P* = 0.0127, unpaired *t* test). (**K**) PhenoTrack analysis shows myeloid depletion restores T cell tumor sampling (*n* = 5, *P* = 0.0369, unpaired *t* test). Error bars represent standard deviation. Source data are available online for this figure.

With TME-CART fully developed, we sought to evaluate immunosuppression of T cells in tumors following myeloid depletion using the DL model trained from the ICB experiment analysis. To reduce myeloid cells, we first treated *KPCT* mice with a small-molecule CCR2 inhibitor (Fig. 7A) that we recently described (Dixit et al, 2022) for two weeks, followed by T cell analysis in tumor slices. Immunofluorescence showed an overall reduction in the number of myeloid cells (Fig. 7B). However, the degree of myeloid cell depletion varied considerably among tumor regions, and one sample showed limited depletion, likely due to the shorter treatment window (2 weeks) used here compared to the greater depletion we previously observed under longer treatments (Dixit et al, 2022). As such, we specifically analyzed regions of strong depletion and incomplete depletion. Indeed, analysis with our DL platform demonstrates that regions of incomplete depletion are associated with higher levels of T cell suppression, whereas in regions of near complete depletion there is much lower T cell suppression (Fig. 7C). Aggregating all DL outputs for the CCR2i treatment, the correlation plot of percentage suppression versus the number of CD11b+ myeloid cells demonstrates a positive correlation trend ($\rho =0.44{;p}=0.0252$) between the presence of residual myeloid cells and T cell suppression (Fig. 7D). These observations confirm that heterogeneity in myeloid depletion is a key factor influencing local T cell behavior.

To generate a more uniform myeloid depletion, we directly treated PDA tumor slices with liposomal clodronate (Fig. 7E). Immunofluorescence shows a strong effect on depleting CD11b+ myeloid cells following treatment with 10% w/v liposomal clodronate, including cases where near-complete depletion was observed, although heterogeneity in the magnitude of depletion remains. Depletion was more uniform than following in vivo treatments (Fig. 7F,G). Consistent with our earlier findings, in control slices meso-TCR T cells were again frequently observed in close spatial proximity to myeloid cells and remained largely confined within the collagen matrix that harbors these myeloid cells (Fig. 7H). In contrast, T cells in myeloid-depleted slices distribute throughout the imaged tumor volumes, even though some T cells still maintain contact with remnant myeloid cells (Fig. 7H). Notably, evaluation of immunosuppression using our DL model demonstrates that meso-TCR T cells in myeloid-depleted groups spend significantly more time in a non-suppressed state compared to controls that have robust myeloid immunosuppression (Fig. 7I). Indeed, entropy estimation using Kozachenko–Leonenko (K-L) k-nearest neighbors (k-NN) entropy estimator of pre- and post-migration T cell coordinates shows a significant increase in dispersity of meso-TCR T cells in myeloid-depleted conditions (Fig. 7J), indicating that the removal of myeloid cells enhances overall T cell dispersal and promotes sampling potential of therapeutic T cells throughout the PDA TME. Applying PhenoTrack, we found that myeloid depletion enhanced T cell sampling, consistent with DL inference results (Fig. 7K). In conclusion, these findings demonstrate that our TME-CART platform can effectively capture non-intuitive immunosuppressive behaviors and quantitatively define the impact of targeting immunosuppressive features.

## Discussion

A key strength of our study lies in the integration of data analytics into biological experiments. The development of the TME-CART pipeline, along with complementary tools such as MechanoTrack, PhenoTrack, and DL models incorporating SHAP, LIME, and PDPs, allowed us to translate high-dimensional imaging data into interpretable insights. Importantly, TME-CART is designed around generic biophysical and behavioral features, rather than PDA-specific biology (e.g., *PhenoTrack* extracts T cell behavioral states from morphological measurements from any time-lapse imaging dataset of immune cells in tissue, *MechanoTrack* quantifies structure-based impedance to T cell infiltration and transforms the ECM into a “terrain map” based on migration anisotropy etc.). Further, these modules accept standard multiphoton or confocal imaging inputs and do not require any PDA-specific parameters, allowing for widespread applicability to cell dynamics. We do note however that a limitation of our approach is the substantial volume of imaging data required to effectively train DL models, which in turn constrains the choice of DL architecture. Although hybrid architectures such as LSTM-FCNN and 3D-CNN could potentially offer deeper insights into temporal dependencies, they demand extensive time series data that is not feasible to collect within a reasonable timeframe due to the requirement of mice and subsequent experiments. As the dimensionality increases, the feature space becomes sparser, and in our testing, these models failed to converge due to the effect of sparse tensors on convolution layers and pooling layers. As a consequence, our DL analysis not only achieved high predictive accuracy (AUC-ROC > 0.97) but also offered mechanistic insights by pinpointing which TME or T cell features most critically modulate T cell behavior. To further validate this architectural choice, we compared the 11-layer FCNN against logistic regression with L2 regularization, linear SVM, and a shallow 3-layer FCNN on both datasets. Nonlinear models consistently outperformed linear approaches, with the 11-layer FCNN achieving the highest accuracy (Table EV2). Notably, the nonlinear models enable the downstream interpretability framework and reveal nonlinear effects and multi-feature interactions. In comparison, logistic regression constrains each feature to a monotonic effect, which would obscure these biologically important nonlinearities. In this context, LIME analysis reveals that engineered meso-TCR T cells are adept at navigating dense collagen regions and that ICB further facilitates their dispersal by distancing them from suppressive myeloid clusters. In addition, two-variable PDPs uncovered how these features synergistically influence T cell behavior. These findings underscore the power of combining DL with biological experiments to unearth complex, nonlinear, and multivariate interactions that would otherwise be difficult to capture with conventional statistical methods alone.

The biophysical problem addressed by TME-CART on ECM and myeloid immunosuppression is not unique to PDA. Collagen-mediated immune exclusion is a defining feature of numerous desmoplastic tumors, which most solid tumors are, where fibrosis and the presence of immunosuppressive cells is robust. Thus, we anticipate wide applicability of our approaches. Our work provides novel insight, via a first-of-its-kind to our knowledge, fibrotic immunosuppressive index. As these architectures, i.e., Tumor-Associated Collagen Signatures (TACS)-like structures, are well established in many solid tumors, and in particular those of the breast, prostate, ovaries, lungs, and colon, we anticipate that our findings will likely inform key T cell – ECM interaction behaviors in these cancers as well. Our findings demonstrate that collagen has a dual role in T cell infiltration within the PDA TME. On one hand, collagen fibers serve as high-affinity highways that promote rapid and directional movement of T cells. On the other hand, the same structural alignment results in migration anisotropy, by which T cells become constrained to predetermined paths, limiting their ability to deviate and effectively search for cognate antigens. The concept of mR, which we quantified computationally, highlights how the collagen network establishes microscopic immune exclusion zones—regions where T cells are energetically discouraged from off-axis movement. This nuanced perspective suggests a balanced approach that sustains the beneficial aspects of directional contact guidance while alleviating the mechanical barriers to effective tumor sampling would be optimal. However, given that the suppressive regions of the collagen network far outweigh the beneficial regions, combined with the highly plastic nature of T cell phenotypes to navigate numerous tissue spaces, it remains to be seen whether moderately depleting collagen would have any benefit over robustly depleting collagen. Likewise, in addition to the potential for altering stromal ECM architectures, our analysis supports the concept of altering T cell adhesion to alleviate aspects of restricted migration. Recent mathematical modeling, validated by experiments, concludes that T cells can undergo motility without adhesion (i.e., swim), but not at consensus speed levels observed in vitro and in vivo, where adhesion is required (Alonso-Matilla et al, 2026). Along these lines, T cells can have very diverse and hybrid phenotypes, and at the receptor level, T cells express key collagen-binding integrins (e.g., α1β1 (CD49a) and α2β1 (CD49b)) as well as other more minor collagen receptors and receptors for cells and ECMs that can colocalize with collagen (Hachehouche et al, 2010; Krummel et al, 2016; Lebbink et al, 2006; Qian et al, 2026; Richter et al, 2007; Tabdanov et al, 2021). This suggests that molecular or cell engineering approaches to reduce these interactions could decrease the trapping and directional migration dependencies that can dominate T cell directional migration.

Beyond the physical barrier posed by collagen, we highlight the critical role of immunosuppressive myeloid cells in further dampening T cell efficacy. Indeed, the spatiotemporal interplay between the myeloid populations and the ECM has not been thoroughly explored, but the work here adds new insights into the pattern of behavior of these interactions. Our data show that myeloid cells colocalize with collagen, forming a dual obstacle that not only fragments the collagen network access paths but also directly interferes with T cell behavior. This heavily reduces available infiltration routes, increases forced tortuosity, as demonstrated using graph theory and Monte Carlo simulations, and promotes myeloid T cell sequestration and exhaustion. The stark contrast in enhanced T cell dispersal and T cell–carcinoma interaction observed following myeloid depletion underscores the pivotal role that these myeloid cells play as cellular gatekeepers within the TME. Furthermore, we note that our current model iteration does not fully capture distance-dependent changes for cells in very close proximity. Identifying the most dominant distance input vectors for distance-dependent chemical communication between a T cell and the immunosuppressive cells is a complex problem, particularly given the number and spatial distribution and the rapid change in these interactions in 4D. Yet, there is a clear need for future imaging and modeling studies to further parse out this complexity. Nevertheless, achieving more uniform myeloid depletion along with disrupting fibrous immunosuppression by concurrently or sequentially altering each component may be part of an essential strategy for maximizing T cell activation and promoting maximally robust anti-tumor responses.

From a translational perspective, our work holds considerable promise for enhancing cell-based immunotherapies for solid tumors. The dual challenge posed by collagen and myeloid cells clarifies why many T cell therapies have faced difficulties in the context of solid tumors. Our data suggest that therapeutic strategies must more thoroughly consider the complex and interconnected/dependent nature of the TME landscape. Thus, engineered T cells (e.g., CAR-T, engineered TCR, TILs, etc.) may benefit from systematic modulation and disruption in both the biophysical (e.g., ECM) and biological aspects (e.g., immunosuppressive myeloid cells) within the TME. Interventions such as focused collagen and myeloid targeting, combined with engineering T cells to be “physically optimized” to move and function better in solid tumors (e.g. (Tabdanov et al, 2021; Zhang et al, 2026)), appear especially promising based on our model outcomes. Likewise, increasing function and longevity will certainly be key factors, in the case of PDA here we note that PDA can become more susceptible to ICB (Dixit et al, 2022; Feig et al, 2013; Jiang et al, 2016). In our case here, we have elevated cell infiltration to study robust T cell dynamics across heterogeneous environments and observe benefit from ICB, suggesting that when sufficient numbers of T cells are present and navigating through a desmoplastic tumor, ICB or deleting surface checkpoint molecules on therapeutic T cells could be beneficial. Yet, we also note that fibrotic immunosuppression remains robust in this context. As such, these insights emphasize the need for a dual approach to have combination stromal targeting therapies to reprogram or ablate the suppressive TME feature, coupled with rationally engineered T cells designed for solid tumor environments. Moving forward, the application of our data-driven methodologies is set to further enhance our understanding of T cell-TME interactions and the design criteria for these focused therapeutic approaches, ultimately paving the way for more effective interventions in PDA and likely other solid tumors characterized by a dense desmoplastic immunosuppressive landscape.

## Methods

**Table:** Reagents and tools table

Reagent/resource	Reference or source	Identifier or catalog number
**Experimental models**		
KPC mice (autochthonous pancreatic ductal adenocarcinoma model)	Hingorani et al, 2005	
TRex mice (T-cell receptor exchange; high-affinity Msln406–414:H-2Db–specific TCR, clone 1045, at the Trac locus)	Rollins et al, 2023	Clone 1045
**Recombinant DNA**		
**Antibodies**		
Anti-CD11b monoclonal antibody, Alexa Fluor 647-conjugated (rat)	Abcam	ab197702-199ul
Anti-H2-Db monoclonal antibody (MHC class I blockade)	Thermo Fisher	MA5-17992
Anti-mouse PD-1 monoclonal antibody (immune checkpoint blockade)	BioXCell	BE0146-A025mg
**Oligonucleotides and other sequence-based reagents**		
**Chemicals, enzymes, and other reagents**		
CellTracker Green cell-labeling dye	Thermo Fisher	C7025
CellTracker Red CMTPX cell-labeling dye	Thermo Fisher	C34552
Dynabeads Mouse T-Activator CD3/CD28	Thermo Fisher	11456D
ImmunoCult-XF T Cell Expansion Medium	STEMCELL Technologies	10981
Recombinant mouse IL-2	STEMCELL Technologies	78081.1
Mouse CD8 + T cell isolation kit (immunomagnetic negative selection)	STEMCELL Technologies	19853
EasySep Mouse CD11b Positive Selection Kit	STEMCELL Technologies	18970
Liposomal clodronate	Encapsula NanoSciences	NC0337390
CCR2 inhibitor (CCX-598)	Dixit et al, 2022	CCX-598
Collagen type I, rat tail	Corning	CB354249
**Software**		
MATLAB R2024a	MathWorks	
Fiji/ImageJ	Schindelin et al, 2012	https://fiji.sc
TME-CARTographer (TME-CART) analysis pipeline	This study	https://github.com/guhanqian/TME-CART
**Other**		
Vibratome	Campden Instruments	
Multiphoton laser-scanning microscope (MPLSM); GaAsP and Multi-Alkali detectors	Bruker	
Live-cell imaging chamber	Warner Instruments	
Organotypic cell-culture inserts, 12 mm	Millipore Sigma	PICM01250

### Live PDA tumor slices

The University of Minnesota Institutional Animal Care and Use Committee (IACUC) approved all animal studies. Animals were maintained in specific pathogen-free conditions at the University of Minnesota Research Animals Resources facility with continual access to food and water and kept on a 12-h light–dark cycle. Experiments conformed to ethical regulations for animal testing and research. Autochthonous PDA tumor is harvested from *KPC/KPCT* mice after reaching 0.8-1 cm by small animal high-resolution ultrasound. Tumor slices were prepared as we have previously described (Rodriguez-Merced et al, 2023; Tabdanov et al, 2021). Briefly, tumor is sliced with a vibratome (Campden Instruments) to a thickness of 300 μm. For cell labeling, tumor slices are placed into 5% normal rat serum in the staining media, RPMI (without phenol red) + L-glutamine+ 1% FBS (200 μL/well), for 1 h in the incubator (37 °C + 5% CO_2_). Tumor slices are stained with an Alexa Fluor 647-conjugated anti-CD11b monoclonal antibody (Abcam) from rats, diluted in 250 μL of staining media containing 5% BSA, for 1 h in the incubator to visualize myeloid suppression. After staining, the tumor tissue was washed and incubated three times in the staining media for 10 min each.

### CD8 + T cell isolation, activation, and expansion

Polyclonal CD8 + T cells were isolated from mouse splenocytes using immunomagnetic negative selection (STEMCELL). Isolated T cells are activated with CD3/CD28 Dynabeads (Thermo Fisher) in ImmunoCult-XF T Cell Expansion Medium (STEMCELL) supplemented with mouse recombinant IL-2 (STEMCELL). Dynabeads are magnetically removed after 24 h, and the CD8 + T cells are ready for use. For therapeutic T cells, tumor-reactive engineered T cells carrying a cloned mesothelin-specific TCR (high-affinity Msln_406-414_:H2D^b^-specific TCR clone 1045) were isolated from T cell receptor exchange (TRex) mice that express the high-affinity Msln_406-414_:H-2D^b^-specific TCR within the physiological *Trac* locus as described (Rollins et al, 2023).

### Multiphoton microscopy of T cell behavior

Multiphoton laser-scanning microscopy (MPLSM) enables multiplexed live cell and tissue volume tracking with combined multiphoton excitation (MPE) and second harmonic generation (SHG) at uncompromised spatiotemporal resolution. As described (Rodriguez-Merced et al, 2023), tumor slices are transferred onto individual collagen-coated (3 mg/mL) organotypic inserts (Millipore Sigma). Activated CD8 + T cells (CD8 + /MSLN-CD8 + ) are stained with CellTracker Green, seeded on top of the tumor slice, and incubated for 1 h for infiltration. After incubation, excess T cells are washed off, and the tumor slice with infiltrated T cells is placed in a 35 mm dish with L-15 + 10% FBS and covered with tissue harp to immobilize the tissue during imaging. For T cell migration experiments with or without Alexa Fluor 647, fluorescence and SHG are excited at 800 nm and 880 nm, respectively, and are collected with Hamamatsu GaAsP (far-red) and Hamamatsu Multi-Alkali detectors (red, green, SHG). Temperature during imaging is controlled through a live cell imaging chamber (Warner Instrument).

### Migration anisotropy coefficient

T cell movement on perfectly aligned substrates containing ICAM-1 provides a controlled environment for extracting the anisotropy coefficient. To obtain the anisotropy coefficient, T cells are segmented with Trainable Weka Segmentation and tracked with TrackMate-Weka in FIJI (Schindelin et al, 2012). The sum of x- and y-movement in pixels is calculated and the coefficient is given as,

$$a=\frac{\sin \left(\pi -\theta \right)\left({\sum}_{i}|{y}_{i}|-{\sum}_{i}|{x}_{i}|\right)+\cos (\pi -\theta )({\sum}_{i}|{x}_{i}|-{\sum}_{i}|{y}_{i}|)}{\sin \left(\pi -\theta \right)\left({\sum}_{i}|{y}_{i}|+{\sum}_{i}|{x}_{i}|\right)+\cos (\pi -\theta )({\sum}_{i}|{x}_{i}|+{\sum}_{i}|{y}_{i}|)}$$

where *θ* is the reference angle (the angle between +x axis and the anisotropy axis) of the nanopattern.

### Mapping of TME mechanoconductance (MechanoTrack)

The collagen networks in the TME imaged by SHG were segmented and analyzed with ridge detection in FIJI. The output statistics were used to compute the collagen-based mR in the TME-CART suite. At each pixel, collagen fiber statistics within a radius were isolated and organized into unique fiber IDs. Based on distance metrics, each unique fiber was assigned a class (i.e., collagen, catch, and resistance) based on the L1 distance. Pixels colocalized within the collagen radius (${r}_{{col}}$) have the lowest collagen-based mR, or the highest mC, $M{C}_{{col}}=1$. Within the catch radius (${r}_{{cat}}$), T cells are free to traverse and catch targets off-collagen but are less probable as distance $d\in ({r}_{{col}},{r}_{{col}}+{r}_{{cat}})$ increases, which is hence modeled by a Gaussian function, $[{r}_{{col}},{r}_{{cat}}]=[{{\arg }}\max G(\mu ,\sigma ),{{\arg }}\min G(\mu ,\sigma )]$, where $\sigma =3{r}_{{cat}}$. Thus, the mC within the sum of mC is influenced by all nearby unique fibers and is thus given by ${MC}=\max (M{C}_{{col}},{\sum }_{i}M{C}_{{col}}G({d}_{i},\sigma ))$. The resistance region is defined as the region outside of the catch radius and is marked by a drastic decrease in mC due to collagen-mediated T cell motility bias, while the end of the fiber opens up space and allows temporary T cell momentum. Thus, mC within this region is simultaneously distance-, angle-, and momentum-modulated. Hence, at each pixel, the relative angle (${\alpha }_{r}$) can be calculated, and the MC is given as ${MC}=\max (-M{C}_{{col}},{\sum }_{i}\left(\frac{|{\alpha }_{r,i}-90|}{90}m-1\right)bG\left({d}_{i},\sigma \right))$. Collagen-deficient regions are considered neutral with ${MC}=0$. Based on these principles, MechanoTrack combines 3D T cell coordinates and mC index at the specific space and time to uncover T cell sampling behavior.

### Myeloid cell isolation and embedding

PDA tumors are harvested from KPC mice after ultrasound confirmation and digested with tissue digestion media (collagenase + hyaluronidase + DNase). Digested tissue is mechanically pressed through a 70-μm cell strainer (Fisher Scientific) using a syringe plunger to further disintegrate the stroma. Using EasySep Mouse CD11b Positive Selection Kit (STEMCELL), CD11b+ myeloid cells in the tumor are immunomagnetically isolated. The isolated myeloid cells are labeled with CellTracker Red CMTPX dye. Stained myeloid cells are then suspended in RPMI + 10% FBS and added to 1:1 stock collagen type I from rat tail (Corning) and HEPES + 2× PBS. The cell-matrix mixture is then transferred to a 24-well plate for polymerization in the incubator (5% CO_2_). After 3 h, each myeloid-embedded collagen gel is topped up with 1 mL of cell culture media (RPMI + 10% FBS).

### Phenotype tracking and pattern recognition of T cell time series (PhenoTrack)

PhenoTrack, a component of the TME-CART algorithm suite, analyzes cell morphology data and velocity to identify the phenotypic behavior of cells. Taking inputs of T cell tracks, T cell morphology, and myeloid cell tracks over time from TrackMate in FIJI, PhenoTrack examines T cell velocity, circularity, the change in cell circularity, and the distance to myeloid cells at each frame for each T cell. Based on tunable thresholds for each parameter, PhenoTrack assigns a plausible status (migration, sensing, repulsion, exhaustion) to each cell at each time point. The time series containing serialized cell status, *S*, are then convolved with a tunable n-element convolutional kernel, ${K}_{n}={\left\{s\right\}}_{i=1}^{n}$, such that for n-consecutive time points, an event is established at t=m if

$$S(t)| t\in [m,m+n]* {K}_{n}={\sum }_{i=m}^{m+n}S{\left(i\right)}^{2}$$

### Undirected graph and Monte Carlo simulation

Collagen fibers are segmented using Ridge Detection (FIJI) and recreated in MATLAB. Macrophages are tracked using TrackMate (FIJI). A mask containing macrophages with blocking radius *r* are drawn and applied to the fiber image. For each fiber pixel, connectivity to nearby fiber pixels is weighted based on distance on a Gaussian membership function (GMF) such that closer fibers have increased weights. Using the connectivity and weight information, we then construct the adjacency matrix and the subsequent undirected graph without self-loops. To obtain the numerical value of path availability, random nodes resembling two points on the collagen fiber network on the undirected graphs are sampled. Using Dijkstra’s algorithm, the shortest path between a pair of nodes is found and compared to the L2 distance. At 10,000 iterations, for the sampled node set *M*, the path availability is thus $P\left({av}.\right)=\frac{\left|M\le \infty \right|}{{|M|}}$, where ∣⋅∣ is the cardinality.

### Deep learning with interpretations

Imaging data are aggregated and pre-processed in MATLAB R2024a (MathWorks). Data aggregation using a custom pipeline assembles spatiotemporal data into large data matrices, which are then processed to generate input matrices containing 23 features for deep learning. Datasets are normalized, zero-centered, and split into training–validation–testing sets (8:1:1). The neural network containing 10 fully connected layers activated by rectified linear unit (ReLU) in a total of 23 layers is trained with stochastic gradient descent with momentum (SGDM), cross-entropy loss, and a scheduled learning rate that decays every 3 epochs from an initial learning rate of 0.001. The model is trained for a total of 8 epochs. Model convergence and testing accuracy are monitored, and early stopping is used to prevent overfitting. The deep learning models are probed by SHAP, LIME, and 1- and 2-variable PDP, respectively, revealing both local interpretations of model predictions and marginal effects resulting from variations in the input features globally.

### Antibody neutralization

To block antigen presentation through MHC Class I, tumor slices were treated with H2-Db monoclonal antibody (clone: B22-249.R1; 20 μg/mL; ThermoFisher) for 1 h. To block PD1/PD-L1 interactions in PDA, T cells in suspension were incubated with anti-mouse PD-1 monoclonal antibody (clone: 29 F.1A12; 90 μg/mL; BioXCell) for 1 h. Cells were washed in L-15 medium supplemented with 1% FBS to eliminate any residual antibodies.

### Depletion of CD11b+ myeloid cells

To deplete CD11b+ myeloid cells from PDA tumor slices, tumor slice culture media (TSCM) was supplemented with 10% w/v liposomal clodronate (Encapsula NanoSciences) for a total of 400 μL of depletion media. Tumor slices were placed on 12 mm organotypic inserts (PICM01250, Millipore Sigma) in a 24-well tissue culture plate. Depletion media (200 μL) was overlaid on top of the tumor slice within the tissue insert, while the remaining 200 μL was pipetted into the plate well outside of the insert to maintain a passive gradient. The treated tumor slices were incubated for 24 h.

To deplete CD11b+ myeloid cells from KPCT mice, a CCR2 inhibitor (CCX-598) was administered daily for 14 days at 100 mg/kg through oral gavage.

### T cell dispersity estimation

T cell dispersity is evaluated using the K-L k-NN entropy estimator. T cell coordinates were tracked using TrackMate and treated as particles with centroids. For a d-dimensional variable, the K-L entropy estimator can be described as

$$\hat{H}=\psi \left(n\right)-\psi \left(k\right)+\log {c}_{d}+\frac{d}{n}{\sum }_{i=1}^{n}\log {\epsilon }_{k}({x}_{i})$$

where *n* is the number of T cells, k=3 is used for defining the neighborhood, ${c}_{d}$ is the volume of the unit ball in ${{\mathbb{R}}}^{d}$, $\psi \left(\cdot \right)$ is the digamma function, and ${\epsilon }_{k}({x}_{i})$ is the L2 distance from *i*-th particle to the *k*th nearest neighbor.

## Supplementary information

Table EV1

Table EV2

Peer Review File

Movie EV1

Movie EV2

Source data Fig. 2

Source data Fig. 3

Source data Fig. 4

Source data Fig. 5

Source data Fig. 6

Source data Fig. 7

Expanded View Figures

## Data Availability

The TME-CART analysis pipeline, including all source code for feature extraction, behavioral phenotyping, fiber connectivity analysis, deep learning classification, and post hoc interpretations, is publicly available on GitHub at https://github.com/guhanqian/TME-CART. Raw experimental data are deposited and available via Sage Bionetwork at www.synapse.org (syn76908324) The source data of this paper are collected in the following database record: biostudies:S-SCDT-10_1038-S44320-026-00243-4.
