# Supplementary material for: Decoding spatiotemporal fibrotic and cellular immunosuppression of therapeutic T cells in live pancreatic ductal adenocarcinoma
Source: Mol Syst Biol. 2026 Sep 10;22(10):1508–30. doi: 10.1038/s44320-026-00243-4 (PMC13639077; doi:10.1038/s44320-026-00243-4)
Supplement: Supplementary file 1 [file 44320_2026_243_MOESM12_ESM.pdf]

Expanded View Figures

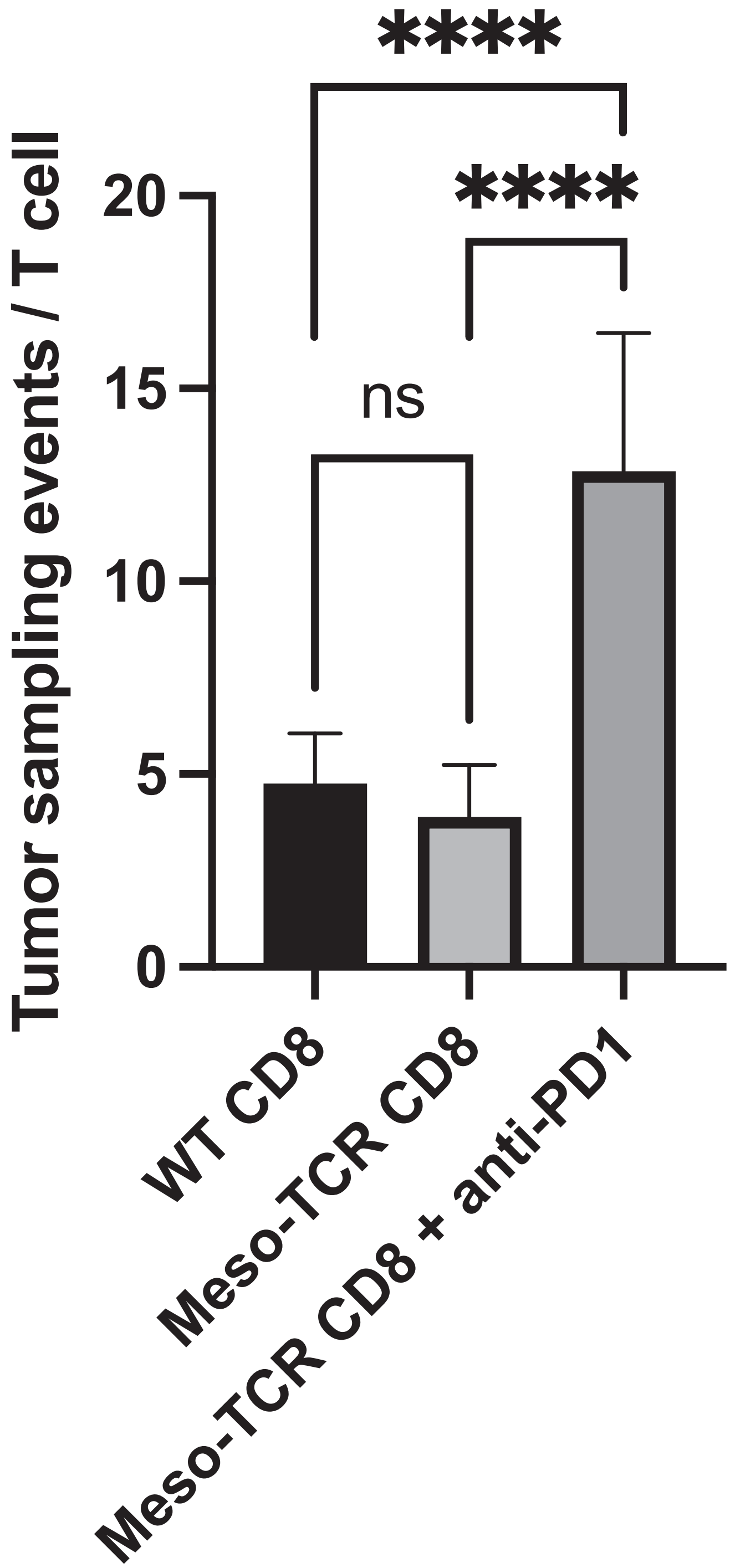

Figure EV1. Anti-PD-1 increases meso-TCR CD8 + T cells tumor sampling compared to WT CD8 + T cells (*n* = 7, ****$P < 0.0001$, one-way ANOVA w/ multiple comparison) and meso-TCR CD8 + T cells alone (*n* = 6, ****$P < 0.0001$, one-way ANOVA w/multiple comparison).

Error bars represent standard deviation.

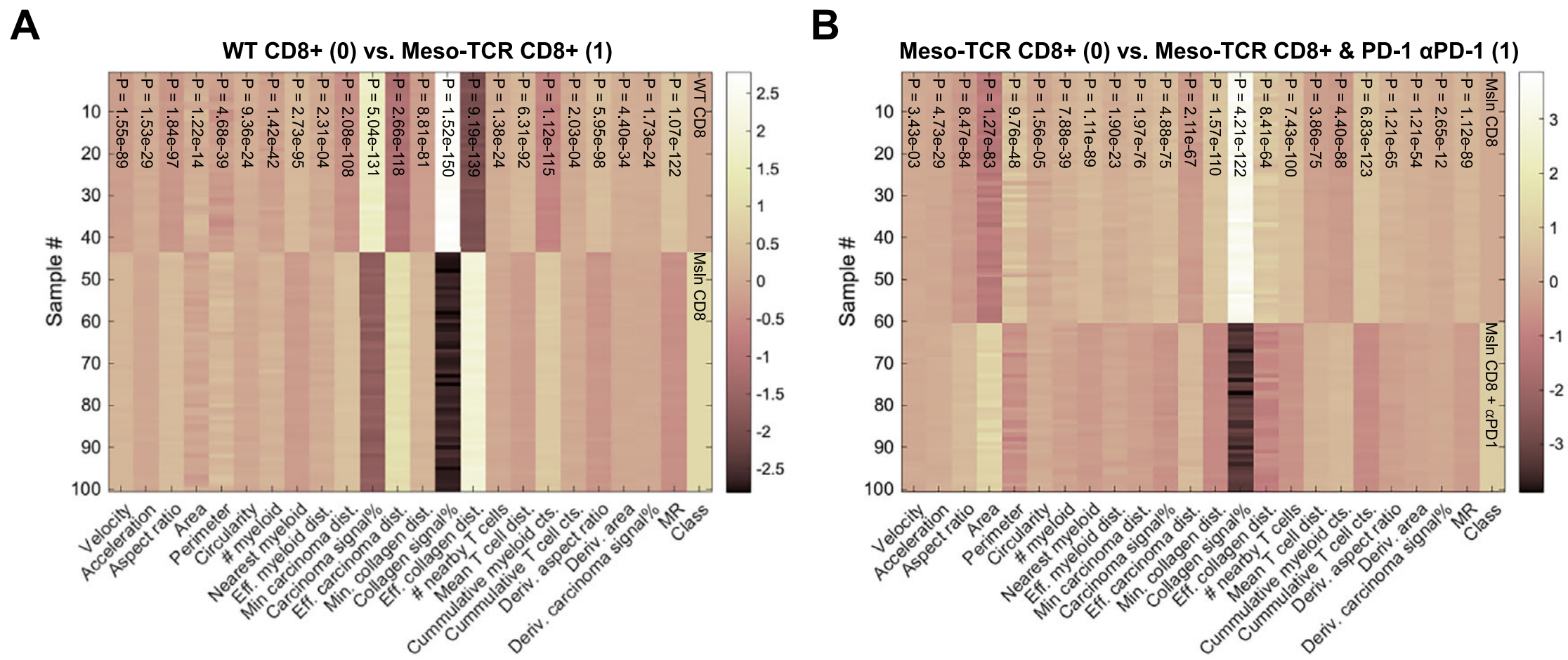

Figure EV2. LIME coefficient analysis.

(**A**, **B**) Aggregate LIME plots of 100 randomly sampled points (WT CD8+ | Meso-TCR CD8 +) and (Meso-TCR CD8+ | Meso-TCR CD8+ with ICB) groups, sorted by class. *P* values show the extent of the difference in mean LIME coefficients between classes.

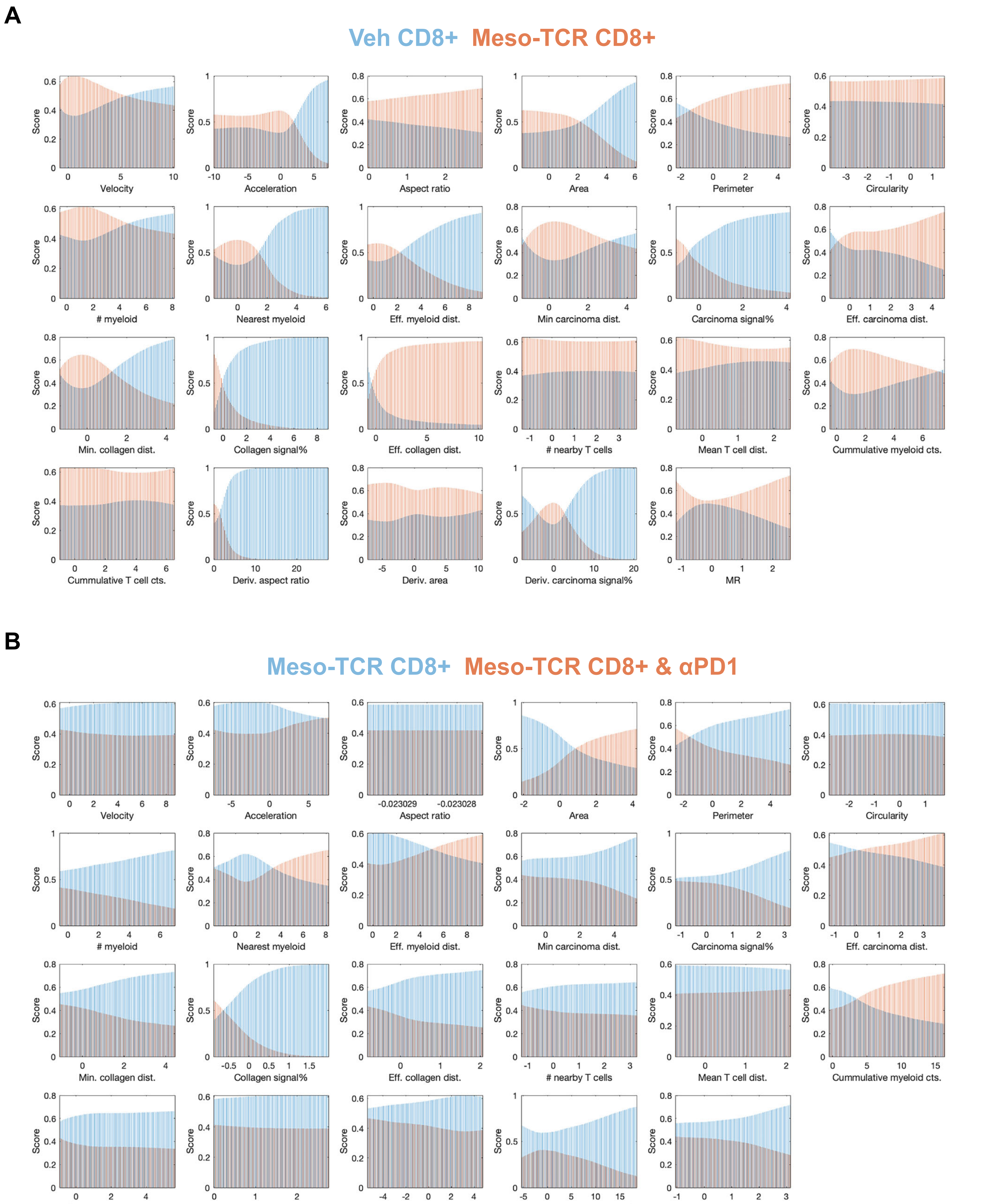

Figure EV3. Partial dependence plots.

(**A**, **B**) Full PDP panels with 23 features included in the DL training (WT CD8+ | Meso-TCR CD8 +) and (Meso-TCR CD8+ | Meso-TCR CD8+ with ICB) experiments.

© The Author(s)
