# Supplementary material for: Decoding spatiotemporal fibrotic and cellular immunosuppression of therapeutic T cells in live pancreatic ductal adenocarcinoma
Source: Mol Syst Biol. 2026 Sep 10;22(10):1508–30. doi: 10.1038/s44320-026-00243-4 (PMC13639077; doi:10.1038/s44320-026-00243-4)
Supplement: Supplementary file 2 [file 44320_2026_243_MOESM3_ESM.pdf]

molecular systems biology

Decoding spatiotemporal fibrotic and cellular immunosuppression of therapeutic T cells in live pancreatic ductal adenocarcinoma

Guhan Qian, Hongrong Zhang, Ingunn M. Stromnes, Kevin Eliceiri, and Paolo Provenzano

Corresponding author(s): Paolo Provenzano (pprovenz@umn.edu)

Review Timeline:

Submission Date:	26th Feb 26
Editorial Decision:	24th Mar 26
Revision Received:	24th Jun 26
Editorial Decision:	16th Jul 26
Revision Received:	10th Aug 26
Accepted:	21st Aug 26

Editor: Jingyi Hou

Transaction Report:

(Note: With the exception of the correction of typographical or spelling errors that could be a source of ambiguity, letters and reports are not edited. Depending on transfer agreements, referee reports obtained elsewhere may or may not be included in this compilation. Referee reports are anonymous unless the Referee chooses to sign their reports.)

24th Mar 2026

Manuscript Number: MSB-2026-13666-T
Title: Decoding spatiotemporal fibrotic and cellular immunosuppression in live pancreatic ductal adenocarcinoma
Author: Paolo Provenzano
Guhan Qian
Hongrong Zhang
Ingunn M. Stromnes
Kevin Eliceiri

Dear Dr Provenzano,

Thank you for submitting your work to Molecular Systems Biology. We have now heard back from the three reviewers who agreed to evaluate your manuscript. Overall, they find the study interesting and novel. However, they have also raised a number of concerns that will need to be convincingly addressed in a major revision.

I think the reviewers' recommendations are relatively clear, so there is no need to reiterate the points listed below. All the issues raised by the reviewers need to be satisfactorily addressed.

As you may already know, our editorial policy allows in principle a single round of major revision so it is essential to provide responses to the reviewers' comments that are as complete as possible. Please feel free to contact me in case you would like to discuss in further detail any of the issues raised by the reviewers.

On a more editorial level, we would ask you to address the following issues:

- Please provide a .docx formatted version of the manuscript text (including legends for main figures, EV figures and tables). Please make sure that the changes are highlighted to be clearly visible.

- Please provide individual production quality figure files as .eps, .tif, .jpg (one file per figure). When assembling figures, please refer to our figure preparation guideline in order to ensure proper formatting and readability in print as well as on screen: https://media.springernature.com/original/springer-cms/rest/v1/content/27825798/data/v1

-Please provide a .docx formatted letter INCLUDING the reviewers' reports and your detailed point-by-point responses to their comments. As part of the EMBO Press transparent editorial process, the point-by-point response is part of the Review Process File (RPF), which will be published alongside your paper.

-Please note that all corresponding authors are required to supply an ORCID ID for their name upon submission of a revised manuscript.

-We replaced Supplementary Information with Expanded View (EV) Figures and Tables that are collapsible/expandable online. A maximum of 5 EV Figures can be typeset. EV Figures should be cited as 'Figure EV1, Figure EV2" etc... in the text and their respective legends should be included in the main text after the legends of regular figures.

Additional Tables/Datasets should be labeled and referred to as Table EV1, Dataset EV1, etc. Legends have to be provided in a separate tab in case of .xls files. Alternatively, the legend can be supplied as a separate text file (README) and zipped together with the Table/Dataset file.

For the figures and tables that you do NOT wish to display as Expanded View figures, they should be bundled together with their legends in a single PDF file called *Appendix*, which should start with a short Table of Content. Each legend should be below the corresponding Figure/Table in the Appendix. Appendix figures and tables should be referred to in the main text as: "Appendix Figure S1, Appendix Figure S2, Appendix Table S1" etc. See detailed instructions regarding expanded view here: https://link.springer.com/journal/44320/submission-guidelines#cms-Expanded-View-data.

-Before submitting your revision, primary datasets (and computer code, where appropriate) produced in this study need to be deposited in an appropriate public database.
Please remember to provide a reviewer password if the datasets are not yet public.
The accession numbers and database should be listed in a formal "Data Availability" section (placed after Materials & Method) that follows the model below (see also https://link.springer.com/partners/embo-press/editorial-policies#Data%20availability%20statement). Please note that the Data Availability Section is restricted to new primary data that are part of this study.
Data availability

The datasets (and computer code) produced in this study are available in the following databases:
- RNA-Seq data: Gene Expression Omnibus GSE46843 (https://www.ncbi.nlm.nih.gov/geo/query/acc.cgi?acc=GSE46843)
- [data type]: [name of the resource] [accession number/identifier/doi] ([URL or identifiers.org/DATABASE:ACCESSION])
*** Note - All links should resolve to a page where the data can be accessed. ***

- At EMBO Press we ask authors to provide source data for the main manuscript figures. You will receive a separate email with instructions for providing source data with your revised manuscript, including how to upload and organize the files.
Additional information on source data and instruction on how to label the files are available <
https://link.springer.com/journal/44320/submission-guidelines#cms-Source-data >

- Our journal encourages inclusion of *data citations in the reference list* to directly cite datasets that were re-used and obtained from public databases. Data citations in the article text are distinct from normal bibliographical citations and should directly link to the database records from which the data can be accessed. In the main text, data citations are formatted as follows: "Data ref: Smith et al, 2001". In the Reference list, data citations must be labeled with "[DATASET]". A data reference must provide the database name, accession number/identifiers and a resolvable link to the landing page from which the data can be accessed at the end of the reference. Further instructions are available at < https://link.springer.com/journal/44320/submission-guidelines#cms-Reference-guidelines >.

- We updated our journal's competing interests policy in January 2022 and request authors to consider both actual and perceived competing interests. Please review the policy (https://link.springer.com/partners/embo-press/editorial-policies#Competing%20interest%20disclosures) and update your competing interests if necessary.
Please use the heading "Disclosure statement and competing interests".

- All Materials and Methods need to be described in the main text using our 'Structured Methods' format. According to this format, the Methods section includes a Reagents and Tools Table (listing key reagents, experimental models, software and relevant equipment and including their sources and relevant identifiers) followed by a Methods and Protocols section describing the methods, ideally using a step-by-step protocol format. The aim is to facilitate adoption of the methodologies across labs.

Please download and fill our Reagents and Tools Table template (.docx), which you can find in our author guidelines: https://link.springer.com/journal/44320/submission-guidelines#structuredmethods.

When submitting your revised manuscript, please do not include the Reagents and Tools Table in the Methods section of the manuscript but upload it as a separate file choosing the file type "Reagent Table".

-Regarding data quantification:
Please ensure to specify the name of the statistical test used to generate error bars and P values, the number (n) of independent experiments (please specify technical or biological replicates) underlying each data point and the test used to calculate p-values in each figure legend. Discussion of statistical methodology can be reported in the materials and methods section, but figure legends should contain a basic description of n, P and the test applied.
Graphs must include a description of the bars and the error bars (s.d., s.e.m.).
Please also include scale bars in all microscopy images.

- Please provide a "standfirst text" summarizing the study in one or two sentences (approximately 250 characters, including space), three to four "bullet points" highlighting the main findings and a " visual abstract" (550px width and 400-600 px height, PNG format) to highlight the paper on our homepage.
Please refer to published papers for examples.

When you resubmit your manuscript, please download our CHECKLIST (https://media.springernature.com/original/springer-cms/rest/v1/content/27825796/data/v1) and include the completed form in your submission.
Please note that the Author Checklist will be published alongside the paper as part of the transparent process .

If you feel you can satisfactorily deal with these points and those listed by the referees, you may wish to submit a revised version of your manuscript. Please attach a covering letter giving details of the way in which you have handled each of the points raised by the referees. A revised manuscript will be once again subject to review and you probably understand that we can give you no guarantee at this stage that the eventual outcome will be favorable.

I look forward to receiving the revised manuscript soon.

Kind regards,
Jingyi

Jingyi Hou, PhD

Senior Editor
Molecular Systems Biology

We realize that it is difficult to revise to a specific deadline. In the interest of protecting the conceptual advance provided by the work, we recommend a revision within 3 months (22nd Jun 2026). Please discuss the revision progress ahead of this time with the editor if you require more time to complete the revisions. Use the link below to submit your revision:

https://msb.msubmit.net/cgi-bin/main.plex

IMPORTANT: Please read our guidelines for revised submissions: https://link.springer.com/journal/44320/submission-guidelines#cms-Revised-submissions

*** PLEASE NOTE *** As part of the EMBO Press transparent editorial process initiative (see our Editorial at https://dx.doi.org/10.1038/msb.2010.72), Molecular Systems Biology publishes online a Review Process File with each accepted manuscripts. This file will be published in conjunction with your paper and will include the anonymous referee reports, your point-by-point response and all pertinent correspondence relating to the manuscript. If you do NOT want this File to be published, please inform the editorial office at contact@molsystbiol.org within 14 days upon receipt of the present letter.

Reviewer #1:

Specific questions to the authors are preceded by '>' below.

Pancreatic cancer still has dismal responses. Most are resistant to ICI therapies, at least partly due to the physical fibrotic nature of the TME. In particular T cells can become stuck in the PDAC storm limiting their ability to engage cancer cells. Additionally a variety of immunosuppressive cells including TAMs and MDSCs limit T cell activity. In mouse models, some T cell constructs show local efficacy but they do not patrol the entire tumor, limiting their clinical benefit. Further defining how T cells interact with their TME could provide insights to enhancing their use clinically.

Here the authors image T cells in live tumors using multi photon microscopy, then use a computational pipeline called TME-CART to understand the temporal behaviors of cells in the TME. They rank features by their predicted ability to modulate T cells and immunosuppression, identifying that eliminate of MDSCs should improve how much of the tumor volume T cells sample.

They use their previously described mouse model of PDAC in which they can follow T cell movement, and which reasonably represents the TME found in human PDAC including collagen and MDSC presence. They combine fluorescent labeling of malignant cells with life IF imaging of target populations of interest such as the T cells.

This platform allows fascinating insight into what T cells are doing in real time, at least in terms of where they are going. Interestingly, T cells preferentially stay around collagen rich regions which can either function as "highway" in specific directions, or to trap them in specific regions. [While carcinoma rich/collagen poor regions have few T cells. This behavior is demonstrated on a nano-pattern substrate which illustrates that TCs tend to follow the collagen "tracks" and are unlikely to cross them.

> Figure 2F is a bit puzzling - what are possible explanations for the bimodal distribution (ignoring the small bump at negative values) of T cell anisotropy ? If it because of the relatively small sample size (n=28) it would be helpful to show the individual points on the plot not just the distribution.

Essentially the TCs tend to follow the path of least resistance through the collagen, which may (or may not) lead to carcinoma cells where they can sample antigens.

Collagen is not the whole story - MDSCs are known to play a role too, and there are many CD11b+ myeloid cells in the PDAC TME as shown. The authors show that over 95% are also located near collagen fibers, but are much slower moving than T cells suggesting they could be roadblocks to the latter. This is supported by engineered TCs for mesothelin which tend to stick around MDSCs in collagen and not sample the tumor volume. Overall the TC behaviors can be characterized as a) sensing b) stuck c) transiting or d) reversing direction; with associated morphologies. This is supported by embedding MDSCs from PDACs in a collagen matrix and observing that migration is decreased and trapping is increased in the presence of the MDSCs.

> The authors mention that this can be due to physical and chemical interactions - this would imply that there is a distance-dependent effect of the MDSCs on TCs. I wonder if it is possible to extract this from the time series data or if it is too coarse?

SInce MDSCs can express high PD-L1 the authors hypothesise that this is a mechanism by which they suppress TC; an alternative is through MHC I interactions. Inhibition of MHCI but not PDL1 appears to inhibit migration, whereas the converse is true for sequestration. Both increase TC repulsion events (though not significantly), but decrease (significantly) myeloid-TC sensing. To support the idea that encountering MDSCs causes TCs to get stuck or take a different route to a target the authors use simulations which support this idea.

> Presumably the TCs that do find a different route do so stochastically, though I wonder if it is possible there is also a "sensing gradient" along the collagen network through secretion from TCs? Or in other words, do the TCs "sense" there is a carcinoma cell nearby and follow the strongest sniff to them where possible?
In relation to this, is the distribution of relative path lengths in the simulations approximate those seen in the experimental set up ? A deviation from this would suggest additional factors, but if they are close it might argue that the MDSCs and collagen physical properties are the predominant factors affecting migration.

The paper then explores other effects on TC migration by building deep learning models in the context of both wild type and meso-specific TCRs, which showed that the latter are better at getting off the collagen network. The DL model indicates there's a "carcinoma signal" in effect here which is what I alluded to above.

> This analysis does also seem to show that mesh-specific TCs are less likely to be associated with carcinoma signal which is the opposite of what I would have anticipated - maybe I am misinterpreting the SHAP plot?

Application of ICBs seems to block this at least partly - wherein TCs in treated conditions are even more likely to go to collagen poor regions implying they are freer to move. IN a local linear analysis to model the DL models and provide better interpretability, again the collagen signal emerges as the strongest influence.

There are various morphological differences between the WT and meso TCs which is interesting, but of course opens up a lot of questions for future investigation.

> Are the PDPs necessary to detect these effects as there is a nonlinear relation, or could they also be obtained by a logistic model? I think this would be valuable in note as many just apply linear models, and this would indicate that this misses important aspects. The two-variable PDPs generally indicate that collagen is the main interaction with other features, with the strongest being myeloid influences. In the presence of anti-PDL1 collagen remains the main factor but the score for myeloid cells decreases, again supporting that these are the two biggest influences on TC migration and that anti-PDL1 impacts the interaction with myeloid cells but not the collagen matrix, which makes sense.

Finally the effect of myeloid cell depletion in PDACs was assessed utilizing a recently described CCR2 inhibitor. The level of TC suppression was correlated (rho=0.44) with the number of myeloid cells depletion in a region

> please provide the p-value for this analysis].

Overall I thought this was a fascinating study which nicely combines computational and experimental approaches. I guess the main question is why ICIs are so ineffective in human PDAC despite their apparent beneficial effect on reducing myeloid cell interactions with TCs, whereas they seem to work quite well here. I guess the difference is the 3D nature of the real tumor which will block any drug from getting to any of the cells, which is another effect of the collagen matrix. In the experiments here the thickness which a drug must penetrate is presumably lower and so they are quite effective. Or perhaps the meso-TCR is such a strong signal that it is a dominant difference between the mouse model and human tumors.
Out of curiosity, I also wonder if there are ways to engineer or bias TCs to avoid myeloid cells which might prompt them to take alternate routes to the carcinoma target?

Minor

It would be helpful to know the timescale of the movies to have some idea how fast cells are moving

The bottom panel of Figure 1 (TME-CART) isn't very informative. Removing it would simplify the figure.

Top of page 8 "employ" and "employed" are redundant in the second sentence.

In the T cell disparity entropy estimator, how is the "number of neighboring T cells" calculated i.e. how is "neighborhood" defined in this context ?

Reviewer #2:

This work describes a an integrated experimental/computational pipeline to characterize the role of collagen in T cell dynamics

in pancreatic ductal adenocarcinoma (PDA). The key conclusions find that collagen can both accelerate T cell activity by providing a mechanical means for the T-cell to find the tumor, but that it can also sequester T-cells from the tumor. The authors used a combination of in vitro systems and computational models to derive this conclusion.

This work seems convincing, though many of the technical details are far outside my area of expertise. While the methodological advances are important, they do not come across as something that can be applied to another system. As such, the main finding of this paper is the understanding of cell types in PDA T cell therapy.

I only have a few comments, primarily about the deep learning model:

1- There is no link to the code, making it difficult to determine precisely how the data was manipulated
2- The neural network is not well described, the authors don't describe how or why the network was built, for example why there were 23 layers. Given that there were only 23 features to evaluate, would it be possible to use a linear approach to get at the same result?
3- There are a few typos, including 'LIME analysis mirrors interpretations concluded using.' and 'in addition to established chemical immunosuppressive signals that can alter T cell behavior (e.g. 45,46).T cell dynamics behavior ...'

Reviewer #3:

Qian et al developed a computational pipeline to investigate T cell-TME interaction in pancreatic cancer (and other solid tumors). The goal is to better define how the TME impacts immune cell trafficking with an eye towards improved immune therapy for pancreatic cancer.

Pancreatic ductal adenocarcinoma (PDA) is a desmoplastic tumor and as such the impact of ECM proteins, including collagen on immune cell trafficking has been of key interest to many groups. The authors image T cells in PDA with multiplexed multiphoton microscopy and analyze the images with TME-CART, a computational discovery pipeline. From their analysis, the authors conclude collagen fibers can function as highways for T cell movement but that it can be difficult for T cells to exit the highway, an effect the authors characterize as T cell migration anisotropy, which can limit T cell recognition of cognate antigens. The authors also highlight that myeloid cells can be important contributors to T cell activity/inactivity.

Strengths of the study:
The displays and the text are clear and generally well-done. Figure 1 provides a cogent overview of the experimental setup that helps orient the reader to the platform and eventual output. The concept that collagen and myeloid cells are key features contributing to limiting T cell interaction with tumor cells is consistent throughout the figures. Figure 2B is particularly easy to interpret and useful.

Minor Comments:
1. The authors highlight that T cells travel on collagen highways. However, it is not clear from the text whether the nature of the interaction of T cells with collagen is known. The authors should discuss potential receptors on T cells that interact with collagen or whether there are other known features that T cells exploit to bind/travel on collagen fibers. Do any of these represent tractable targets to improve T cell sensing of tumor cells?

2. Figure 3D: collagen is not apparent or at least is difficult to discern in the images.

3. Myeloid cell phenotype is central to the story presented. The authors focus on anti-inflammatory TAMs. Do myeloid cells that have a pro-inflammatory phenotype also appear restricted to collagen networks? If myeloid cell phenotype is altered (rather than depletion of myeloid cells) would they interact with T cells via collagen networks?

4. I found Figure 6A/B difficult to interpret. Potentially simplifying or highlighting key findings might improve delivery of the message?

5. Top line of page 9. 'Overall, the LIME analysis...' This is an incomplete sentence.

Response to Reviewer #1

We thank Reviewer 1 for the thorough and insightful evaluation of our work. We appreciate the recognition that this study nicely combines computational and experimental approaches. We address each point below. Reviewer comments are shown in ***bold italic***, and our responses follow in regular text.

"Figure 2F is a bit puzzling — what are possible explanations for the bimodal distribution (ignoring the small bump at negative values) of T cell anisotropy? If it is because of the relatively small sample size (n=28) it would be helpful to show the individual points on the plot not just the distribution."

We thank the reviewer for this observation and for the discussion it raises regarding the modes of contact guidance within T cell behavioral subtypes. In the revised **Fig. 2E**, we have overlaid the individual data points on the distribution to provide direct visualization of the underlying sample (n=28).

On the bimodality of the migration anisotropy coefficient, the two peaks likely reflect two distinct modes of contact guidance. The mode at ~0.5, which is driven by the majority of the cells, corresponds to T cells persistently tracking along the nanopattern groove, whereas the mode at ~0.1 corresponds to a smaller number of cells undergoing intermittent contact guidance and alternating between groove-aligned migration and exploratory lateral migration. While both peaks are within the range of migrating along the pattern ($\alpha>0$), it is important to note that here the groove width is 800nm, which is much smaller than the diameter of a T cell (~10μm), and the T cells can readily sense and traverse at an angle in some instances in time.

Additionally, the separation of two peaks is consistent with the heterogeneous and discrete motility states of T cells that differ in behavioral and functional phenotypes. For instance, a previous study of T cell timelapse using machine learning has identified "scanner" and "sprinter" subpopulations of T cells[1], consistent with our own data (manuscript in preparation), and literature showing T cells having migration interrupted by phases of sampling, which supports the notion that T cells occupy functionally distinct migratory states or intermittent behaviors. As such, it is interesting that the migration anisotropy coefficient, rather than following a unimodal Gaussian distribution, has indeed or perhaps fortuitously captured such underlying phenotypic distinctness.

The small bump at negative values likely represents T cells that transiently move perpendicular to the alignment axis, which we have also seen in live tumors as rare off-axis travel events.

"The authors mention that this can be due to physical and chemical interactions — this would imply that there is a distance-dependent effect of the MDSCs on TCs. I wonder if it is possible to extract this from the time series data or if it is too coarse?"

We thank the reviewer for this excellent point. We agree that myeloid suppression of T cells in general involves both contact-dependent mechanisms (e.g., PD-L1/PD-1 engagement etc.) and paracrine mechanisms that act over longer scales than direct cell-cell interactions, but still quite short in terms of cell scale distances (often ~10-

100microns)[2]. Our analyses currently show that T cell behavior is strongly influenced by the local collagen-myeloid neighborhood and that myeloid cells act as roadblocks within collagen-rich stroma. Unfortunately, in our current iteration it is not possible for us to fully separate physical interactions and distance-dependent changes for cells in very close proximity, particularly since the myeloid cells are frequently so great in number and densely clustered in regions of the tumor. This creates great difficulty in identifying the most dominant distance input between the T cell and the immunosuppressive cells, particularly given the rapid change in these interactions in 4D. We do, however, build several distance factors into our analysis to capture both contact-scale and neighborhood-scale relationships. For instance, we compute (1) the nearest T cell-myeloid cell distance, which reflects the closest potential site of physical interaction; (2) the number of myeloid cells within a tunable 3D local neighborhood; and (3) a Gaussian-weighted "effective" distance, where the "contribution" of each myeloid cells decays smoothly with distance. Moreover, in our perturbation studies with checkpoint and MHC interaction inhibition we show that disrupting the physical interactions has a profound influence on T cell behaviors, but does not significantly influence repulsion events. We have added text to the Discussion section to discuss this in and the need for future studies to further parse out this complexity.

"Presumably the TCs that do find a different route do so stochastically, though I wonder if it is possible there is also a 'sensing gradient' along the collagen network through secretion from TCs? Or in other words, do the TCs 'sense' there is a carcinoma cell nearby and follow the strongest sniff to them where possible? In relation to this, is the distribution of relative path lengths in the simulations approximate those seen in the experimental set up? A deviation from this would suggest additional factors, but if they are close it might argue that the MDSCs and collagen physical properties are the predominant factors affecting migration."

We thank the reviewer for this question. Overall, we don't observe consistent patterns of T cell leader-follower behavior, suggesting limited secretion from leader cells along the matrix. We would also like to clarify that T cell navigation in the TME is unlikely to be purely stochastic in the sense of an unconstrained random walk. Our data support a model where T cells move within an intricate matrix that is defined by the collagen architecture. With regard to TCs sensing carcinoma cells nearby, we agree with reviewer regarding chemokine gradients from various directions combining into an effective gradient vector, representing the "strongest sniff". Our data suggest that this effective often gradient competes with other incentives, and in this study, we have demonstrated that the ECM architecture in the PDA TME most frequently, but not exclusively, overpowers other gradients. With regard to path lengths in the simulations, in the simulated myeloid depletion, the graphs are constructed directly from tumor ECM structure from SHG of *KPCT* tumor data with the same relative scale, and is thus true to the experimental setup and the PDA pathophysiology.

"This analysis does also seem to show that meso-specific TCs are less likely to be associated with carcinoma signal which is the opposite of what I would have anticipated — maybe I am misinterpreting the SHAP plot?"

We thank the reviewer for pointing out the SHAP plot. The key point here is that the SHAP plot does not directly measure productive T cell-carcinoma engagement. In this case, the carcinoma signal percentage (%) represents the proportion of local carcinoma fluorescence signal at each time point. Therefore, a lower carcinoma signal % means that meso-TCR T cells are more often found in local neighborhoods with lower percentage carcinoma fluorescence than WT T cells (i.e. increased sampling of all regions and regions with sparse carcinoma cells). This is likely in part due to less sampling constraints from the collagen networks, but we note that the T cells are still experiencing immunosuppressive influence from myeloid cells that can be reduced through ICB (now shown in Fig. EV3, and consistent with Figure 4 where PhenoTrack analysis captures increase tumor sampling behaviors following addition of ICB). We have edited the text to better describe the data and reflect the possible implications.

"Are the PDPs necessary to detect these effects as there is a nonlinear relation, or could they also be obtained by a logistic model? I think this would be valuable to note as many just apply linear models, and this would indicate that this misses important aspects."

We thank the reviewer for this important methodological question which drove additional analysis that is now included in the revised manuscript. The nonlinear relationships revealed by the PDPs (i.e Fig. 6) cannot be recovered from a standard logistic regression. Linear approaches like logistic regression, by design, constrain each feature to a monotonic effect with either a positive or a negative coefficient. This could be potentially misleading in that it would suggest that an increase in a feature value would have the same impact on the prediction of the model across the entire domain. The PDPs instead show, for example, that meso-TCR T cells can be associated with both high and low mechanoresistance (MR), but not average MR, or that collagen signal percentage saturates rather than increasing linearly. These nonlinear patterns are inherently important to the description of the true feature dynamics, where nonlinearity is expected.

Inspired by the Reviewer's comment, to further show that linear models are insufficient, we compared a logistic regression with L2 regularization, a linear support vector machine (SVM), and a shallow fully connected neural network (FCNN) on the same datasets used in the study. The analyses (including below and in the new Table EV2 in the revision) show that, in both datasets, nonlinear models consistently outperformed linear models and the 11-layer NNs used in our original manuscript outperformed the shallow 3-layer NNs, indicating that nonlinearity and depth are both required for model performance.

Dataset – WT CD8+ vs. Meso-TCR CD8+

Model	AUC-ROC (0-1)	Testing accuracy (0-1)	Linear (Y/N)
**FCNN-(11-layer)***	**0.9851***	**0.9265***	N*
Shallow FCNN (3-layer)	0.9383	0.8703	N
Logistic Regression w/ L2 regularization	0.8320	0.7884	Y
Linear SVM	0.8304	0.7887	Y

*used in the study

Dataset – Meso-TCR CD8+ vs. Meso-TCR CD8+ & aPD-1

Model	AUC-ROC (0-1)	Testing accuracy (0-1)	Linear (Y/N)
**FCNN-(11-layer)***	**0.9726***	**0.9420***	N*
Shallow NN (3-layer)	0.9693	0.9031	N
Logistic Regression w/ L2 regularization	0.9210	0.8376	Y
Linear SVM	0.9210	0.8409	Y

*used in the study

Beyond classification performance, a primary motivation for our neural network approach is the interpretability framework (SHAP, LIME, 1-var PDPs, and 2-var PDPs), as the PDPs derived from the DNNs reveal biologically meaningful nonlinear relationships including biphasic effects for mechanoresistance, saturation effects for collagen signal, and multi-feature interaction patterns, etc., all of which cannot be recovered from a linear model.

Here, we also note that in the Meso-TCR CD8+ vs. Meso-TCR CD8+ & aPD-1 dataset, linear models performed better, compared to the WT CD8+ vs. Meso-TCR CD8+ dataset. This is consistent with the biological expectation that aPD-1 treatment produces a more uniform shift in the feature space, which makes it more linearly separable. In contrast, the distinction between WT and TCR T cells involves more heterogeneous changes in the feature space, which resulted in a more complex decision boundary that linear models cannot adequately capture. This differentiation is also partly captured with the 2-var PDPs, where the WT CD8+ vs. Meso-TCR CD8+ group produced shifts that are larger in amplitude and quantity, suggesting more complex multivariate interactions exist.

In the revised manuscript, we have modified the Discussion and Supplement sections to include the model comparison and the rationale of using a nonlinear DL model.

Text added in the Discussion:

"…To further validate this architectural choice, we compared the 11-layer FCNN against logistic regression with L2 regularization, linear SVM, and a shallow 3-layer FCNN on both datasets. Nonlinear models consistently outperformed linear approaches, with the 11-layer FCNN achieving the highest accuracy (**Table EV2**). Notably, the nonlinear models enable the downstream interpretability framework and reveal nonlinear effects and multi-feature interactions. In comparison, logistic regression constrains each feature to a monotonic effect, which would obscure these biologically important nonlinearities…"

"Regarding the correlation of T cell suppression with myeloid cell depletion, ρ = 0.44] please provide the p-value for this analysis."

We thank the reviewer for the comment and have updated the text, figure text, and Fig. 7D to include the p value (ρ=0.44, p=0.0252).

"I guess the main question is why ICIs are so ineffective in human PDAC despite their apparent beneficial effect on reducing myeloid cell interactions with TCs, whereas they seem to work quite well here."

appreciate the translational question. We note that PDA is a notoriously immunologically "cold tumor" with little T cell priming and limited infiltration. As such, there is not a large

pool of reactive T cells to fight the tumor. However, when stromal or myeloid targeting therapies are employed in pre-clinical models, PDA can become more susceptible to ICB (e.g. PMIDs: 27376576, 36256464, 24277834). In our case here, we have elevated cell infiltration to study robust T cell dynamics across heterogeneous environments. In this context we do see benefit for T cell dynamics for TCR T cells through reduced interactions with myeloid cells, suggesting an actionable approach to overcoming one aspect of resistance when a robust priming has in fact taken place or an expanded therapeutic T cell adoptive cell transfer approach is employed. However, we note that fibrotic immunosuppression remains robust. Ultimately, we believe it is likely that multiple components of the stroma will need to be normalized, likely along with T cell that are engineered to function more robustly in solid tumors, to overcome suppression. We have edited text in the Discussion to reflect this point.

"Out of curiosity, I also wonder if there are ways to engineer or bias TCs to avoid myeloid cells which might prompt them to take alternate routes to the carcinoma target?"

We thank the reviewer for the interesting question, and this is indeed an exciting translational direction. This is an ongoing research effort in our program, informed in part by the models and analysis presented here. We are exploring approaches to make them less guided by the ECM architecture and also approaches that limit interactions with immunosuppressive cells (both physically and chemically). We have edited text in the Discussion to reflect this. Overall, our approach may give insight into ways to make cell engineering design decisions, and TME-CART is well suited to help test such strategies by quantifying myeloid encounters, T cell-carcinoma cell interaction events, sequestration events, route choices etc.

Minor Comments

"It would be helpful to know the timescale of the movies to have some idea how fast cells are moving."

We agree that knowing the timescale is important context. We have labelled all supplement videos with timestamps in hour:minute:second format and a scalebar. The video playback is at 7 frames per second.

"The bottom panel of Figure 1 (TME-CART) isn't very informative. Removing it would simplify the figure."

We appreciate this suggestion and agree that, for readers already familiar with analysis workflows in the study, the bottom panel is not strictly necessary. However, in presenting this work to broader audiences, in particular towards cancer biologists and immunologists, who may not routinely work with these computational pipelines, we have consistently found that the bottom panel to be a valuable orientation to the workflow, providing a compact map between the experimental inputs and the downstream analytical modules. Thus, we would prefer to retain the bottom panel for a broad audience.

"Top of page 8: 'employ' and 'employed' are redundant in the second sentence."

The sentence with the redundancy has been corrected in the revised manuscript, and we have re-proofread the manuscript to catch typographic and grammatical errors.

"In the T cell disparity entropy estimator, how is the 'number of neighboring T cells' calculated, i.e. how is 'neighborhood' defined in this context?"

We thank the reviewer for this question and wish to clarify how neighborhood is defined in the KL entropy estimator.

$$\hat{H} = \psi(n) - \psi(k) + \log c_d + \frac{d}{n}\sum_{i=1}^{n} \log \epsilon_k(x_i)$$

Here, k specifies the number of neighboring T cells and in the study, we use $k = 3$. We chose $k = 3$ as the neighborhood size to balance the sensitivity to the T cell heterogeneity. A small neighborhood size, such as $k = 1$ (meaning each cell is its own neighborhood), can be too sensitive or unstable, while a neighborhood size too large would smooth out estimates at the cost of sensitivity to local structure.

For each T cell centroid x_i, L2 distances to other T cell centroids were computed and ranked. The distances are defined by the $\epsilon_k(x_i)$ term. This means that the estimator uses a fixed number of neighbors but an adaptive spatial radius which is rank-based and can expand in T cell-sparse regions and contracts in T cell-dense regions. The radius adaptability is key to address the heterogeneous T cell distributions typical of PDA immune neighborhoods.

We have clarified the neighborhood k used in the study in the revised Methods section.

Response to Reviewer #2

We thank Reviewer 2 for the supportive evaluation and for the constructive feedback, particularly regarding the question of broader applicability. We address each point below. Reviewer comments are shown in ***bold italic***, and our responses follow in regular text.

"While the methodological advances are important, they do not come across as something that can be applied to another system. As such, the main finding of this paper is the understanding of cell types in PDA T cell therapy."

We appreciate the reviewer's assessment and agree that the primary biological insights of this study are centered on PDA. However, we would like to respectfully clarify the broader applicability of the TME-CART platform.

The biophysical problem addressed by TME-CART on ECM and myeloid immunosuppression is not unique to PDA. Collagen-mediated immune exclusion is a defining feature of numerous desmoplastic tumors, which most solid tumors are, where fibrosis and presence of immunosuppressive cells is robust. Thus, we anticipate wide applicability of our approaches. Furthermore, the spatiotemporal interplay between the

myeloid populations and the ECM has not been thoroughly explored, but the work here adds new insights into the pattern of behavior of these interactions. Our work provides novel insight, via a first of its kind to our knowledge, fibrotic immunosuppressive index. As these architectures, i.e. TACS-like structures, are well established in many solid tumors, and in particular those of the breast, prostrate, ovaries, lungs, and colon, we anticipate that our findings will likely inform key T cell – ECM interaction behaviors in these cancers as well.

Secondly, the computational modules within TME-CART are designed around generic biophysical and behavioral features, rather than PDA-specific biology. For example, *PhenoTrack* extracts T cell behavioral states from morphological measurements from any timelapse imaging dataset of immune cells in tissue. *MechanoTrack* quantifies structure-based impedance to T cell infiltration and transforms the ECM into a "terrain map" based on migration anisotropy. All these modules accept standard multiphoton or confocal imaging inputs and do not require any PDA-specific parameters. The DL + post hoc interpretability framework (SHAP, PDP, LIME) similarly operates on the extracted feature space, which should be carefully designed to be biologically relevant, and may be retrained on data from any tumor type, with the expectation that the relative feature rankings could shift in informative ways.

Additionally, the T cell behavioral states we have defined are not PDA-specific phenomena. T cell sequestration by myeloid cells, contact guidance-based migration along fibers, and antigen-directed sensing are fundamental behaviors observed across solid tumors. TME-CART provides a quantitative framework for measuring and comparing these behaviors, enabling, for example, a direct comparison of how effectively engineered T cells patrol collagen-rich vs. collagen-poor tumors across different cancer types.

We acknowledge that the current manuscript does not sufficiently emphasize the applicability to other systems. To further enhance adaptability, we have extensively commented and prepared the code to facilitate adoption to other imaging modalities and cell types. We have also added text to the Discussion to highlight these key points.

"There is no link to the code, making it difficult to determine precisely how the data was manipulated."

We appreciate the reviewer's comment on the code availability. The TME-CART codebase was submitted as a code capsule with the original manuscript. However, we recognize that a publicly accessible repository is the standard expectation. The complete TME-CART code has now been deposited in GitHub [https://github.com/guhanqian/TME-CART]. The repository includes documentation and instructions for reproducing all analyses, and the datasets will be available upon request. This information has been added to the Data Availability section of the revised manuscript.

"The neural network is not well described, the authors don't describe how or why the network was built, for example why there were 23 layers."

We thank the reviewer for this comment and acknowledge that the network architecture was insufficiently described in the original submission.

In the revised manuscript, we added the following description of the neural network architecture to the Method section and modified the figures to reflect the changes.

"Our network consists of 11 fully connected layers (10 hidden layers + 1 output layer) with ReLU activation, followed by a softmax classification layer. There are 23 layers in total with 11 weighted layers. The hidden layers use a funnel architecture with 1000 neurons followed by layers of 100 and 10 neurons, narrowing to a binary classification output. The layer compression forces the network to learn increasingly abstract representations of the feature space. Compared to a linear model, the DNN enables downstream post hoc interpretive tools to extract nonlinear feature dynamics."

"Given that there were only 23 features to evaluate, would it be possible to use a linear approach to get at the same result?"

We thank the reviewer for this important question. To directly address whether a linear model could achieve comparable results, which is also in line with a question from Reviewer 1, we trained a logistic regression with L2 regularization model, a linear support vector machine (SVM), and a shallow fully connected neural network (FCNN) on the same datasets used in the study. The analyses show that, in both datasets, nonlinear models consistently outperformed linear models and the 11-layer NNs used in our original manuscript outperformed the shallow 3-layer NNs. This indicates that nonlinearity and depth are both required for model performance.

Beyond classification performance, the primary motivation for our neural network is the interpretability framework (SHAP, LIME, 1-var PDPs, and 2-var PDPs). PDPs derived from the DNNs reveal biologically meaningful nonlinear relationships including biphasic effects for mechanoresistance, saturation effects for collagen signal, and multi-feature interaction patterns, etc., all of which cannot be recovered from a linear model. Linear approaches like the logistic regression, by design, constrains each feature to a monotonic effect with either a positive or a negative coefficient. This could be potentially misleading in that it would suggest that an increase in a feature value would have the same impact on the prediction of the model across the entire domain. The PDPs instead show, for example, that meso-TCR T cells are associated with both high and low mechanoresistance (MR), but not average MR, or that collagen signal percentage saturates rather than increasing linearly. These nonlinear patterns are inherently important to the description of the true feature dynamics, where nonlinearity is expected.

Here, we also note that in the Meso-TCR CD8+ vs. Meso-TCR CD8+ & aPD-1 dataset the linear models performed better than they did when applied to the WT CD8+ vs. Meso-TCR CD8+ dataset. This is consistent with the biological expectation that aPD-1 treatment produces a more uniform shift in the feature space, which makes it more linearly separable. In contrast, the distinction between WT and antigen-specific T cells involves more heterogeneous changes in the feature space, which resulted in a more complex decision boundary that linear models cannot adequately capture. This differentiation is also partly captured with the 2-var PDPs, where the WT CD8+ vs. Meso-TCR CD8+ group produced shifts that are larger in amplitude and quantity, suggesting more complex multivariate interactions exist.

In the revised manuscript, we have modified the Discussion and Supplement sections to include the model comparison and the rationale of using a nonlinear DL model.

Added in Discussion:

"To further validate this architectural choice, we compared the 11-layer FCNN against logistic regression with L2 regularization, linear SVM, and a shallow 3-layer FCNN on both datasets. Nonlinear models consistently outperformed linear approaches, with the 11-layer FCNN achieving the highest accuracy (**Table EV2**). Notably, the nonlinear models enable the downstream interpretability framework and reveal nonlinear effects and multi-feature interactions. In comparison, logistic regression constrains each feature to a monotonic effect, which would obscure these biologically important nonlinearities."

Added in Supplement:

Dataset – WT CD8+ vs. Meso-TCR CD8+

Model	AUC-ROC (0-1)	Testing accuracy (0-1)	Linear (Y/N)
**FCNN-(11-layer)***	**0.9851***	**0.9265***	N*
Shallow FCNN (3-layer)	0.9383	0.8703	N
Logistic Regression w/ L2 regularization	0.8320	0.7884	Y
Linear SVM	0.8304	0.7887	Y

*used in the study

Dataset – Meso-TCR CD8+ vs. Meso-TCR CD8+ & aPD-1

Model	AUC-ROC (0-1)	Testing accuracy (0-1)	Linear (Y/N)
**FCNN-(11-layer)***	**0.9726***	**0.9420***	N*
Shallow NN (3-layer)	0.9693	0.9031	N
Logistic Regression w/ L2 regularization	0.9210	0.8376	Y
Linear SVM	0.9210	0.8409	Y

*used in the study

"There are a few typos, including 'LIME analysis mirrors interpretations concluded using.' and 'in addition to established chemical immunosuppressive signals that can alter T cell behavior (e.g. 45,46).T cell dynamics behavior …'"

We thank the reviewer for catching these errors. Both have been corrected in the revised manuscript. The manuscript has been proofread again to catch typographic errors.

(a) The incomplete sentence now reads: "Overall, the LIME analysis largely mirrors interpretations concluded using SHAP."

(b) The second sentence now reads "Together, these data indicate that, in addition to established chemical immunosuppressive signals (e.g.[3,4]), direct contact with myeloid cells can also influence T cell behaviors."

Response to Reviewer #3

We thank Reviewer 3 for the thoughtful and constructive feedback. We address each point below. Reviewer comments are shown in ***bold italic***, and our responses follow in regular text.

"The authors highlight that T cells travel on collagen highways. However, it is not clear from the text whether the nature of the interaction of T cells with collagen is known. The authors should discuss potential receptors on T cells that interact with collagen or whether there are other known features that T cells exploit to bind/travel on collagen fibers. Do any of these represent tractable targets to improve T cell sensing of tumor cells?"

> We thank the reviewer for the comment and agree that having the context of T cell-collagen interaction at the receptor level will add to our conclusions. We therefore revised the Discussion to distinguish two aspects of T cell interactions in PDA. We highlight factors that are known to regulate T cell adhesion, noting that collagen fiber can set architecture and also have other ECMs integrated or bound. We also comment on no adhesion vs. low adhesion behaviors in the Discussion section.

"Figure 3D: collagen is not apparent or at least is difficult to discern in the images."

> We appreciate the reviewer for noting the low collagen contrast in Fig. 3D. To address this, we re-adjusted the display range of the collagen (SHG) channel for illustration only so that the dense collagen separating the collagen-myeloid complex and the carcinoma-rich zone is clearly visible. The revised Fig. 3D has been updated accordingly.

"Myeloid cell phenotype is central to the story presented. The authors focus on anti-inflammatory TAMs. Do myeloid cells that have a pro-inflammatory phenotype also appear restricted to collagen networks? If myeloid cell phenotype is altered (rather than depletion of myeloid cells) would they interact with T cells via collagen networks?"

> We thank the reviewer for raising this important point on various subtypes of TAMs and their potential interactions. CD11b+ cells in human PDA and the KPC PDA model used in the study are predominantly (over 95%) myeloid TAMs and MDSCs that predominately display immunosuppressive phenotypes, while classically activated macrophages are extremely sparse are relatively sparse (Elahi-Gedwillo et al. 2019, Elyada et al., 2019, Dixit et al., 2022). Thus, we do not isolate this rare population in our analysis, and our quantitative analyses reflect the dominant suppressive population.
>
> That said, this is a very interesting question, since classically activated macrophages do not display the cell shape phenotypes we more robustly observe for TAMs associated with collagen networks (Callaway et al., Matrix Biology 2025), we suspect they may be less directed by the collagen network, suggesting that shifting phenotype, or perhaps even polarization state, could be an interesting strategy to alter TAM behaviors. However, we note that we have also observed that primary tumor-isolated TAMs can differ in behavior from macrophages polarized in vitro, and the rarity creates issues with isolating the rare classically activated population from tumors, so this is quite speculative at this stage. However, we have brief added text to Results on this point.

"I found Figure 6A/B difficult to interpret. Potentially simplifying or highlighting key findings might improve delivery of the message?"

We agree and thank the reviewer for the feedback on message delivery. We re-structured Fig. 6 with captions for each set of PDPs to highlight key findings and redirected the rest of the panel to the Supplement for improved clarity.

"Top line of page 9. "Overall, the LIME analysis…" This is an incomplete sentence."

We thank the reviewer for catching the typographic errors. We have proofread the manuscript again and fixed similar typos. For this sentence, now it reads "Overall, the LIME analysis largely mirrors interpretations concluded using SHAP."

16th Jul 2026

Manuscript Number: MSB-2026-13666R
Title: Decoding spatiotemporal fibrotic and cellular immunosuppression in live pancreatic ductal adenocarcinoma
Author: Guhan Qian
Hongrong Zhang
Ingunn M. Stromnes
Kevin Eliceiri
Paolo Provenzano

Dear Dr Provenzano,

Thank you for sending us your revised manuscript. We have now heard back from the three reviewers who were asked to re-evaluate your study. As you will see below, the reviewers are now satisfied with the modifications made. Before we can formally accept your manuscript, we would ask you to address the following issues:

1. The minor comments from Reviewer #2.

On a more editorial level:

1. Remove "Short running title: Decoding immunosuppression in solid tumors" from the manuscript text.

2. The declaration of competing interests statement should be given a section title, "Disclosure and competing interests statement," and moved from the title page to a position below the "Acknowledgements."

3. The references need to be formatted according to the Molecular Systems Biology reference style. Please list up to 10 co-authors of a paper before adding et al. in the reference list. Citations should be listed in alphabetical order.

4. Please provide a "standfirst text" in a separate DOCX file summarizing the study in one or two sentences (approximately 250 characters, including space), three to four "bullet points" highlighting the main findings.

Please also provide a visual abstract as a separate PNG file with dimensions of exactly 550 px (width) × 300 px (height) to highlight the paper on our homepage.
Please refer to published papers for examples.

5. The two movie files should be renamed Movie EV1 and Movie EV2, and all corresponding callouts in the manuscript should be updated accordingly. The movie legends should be removed from the manuscript and provided separately in the ZIP file together with their respective movie files.

6. The Supplementary Tables should be renamed Table EV1 and Table EV2, respectively, and provided in .xlsx format. The table legends should be removed from the manuscript and included above their corresponding tables.

7. Please add missing callouts for Fig. 7E and individual panels of EV figures.

8. Data availability: According to our data availability guidelines, the imaging datasets underlying this study should be deposited in an appropriate public repository prior to publication. To facilitate the revision process, please include a statement in the Data Availability section indicating where you've deposited the data. The repository accession number and specific URL can be provided at the proof stage once it becomes available. Please remove "Datasets are available upon request".

9. Please download and fill our Reagents and Tools Table template (.docx), which you can find in our author guidelines: https://link.springer.com/journal/44320/submission-guidelines#structuredmethods.

When submitting your revised manuscript, please do not include the Reagents and Tools Table in the Methods section of the manuscript but upload it as a separate file choosing the file type "Reagent Table".

10. Author cheklist: please enter corresponding author name, journal name and manuscript number.

11. Our routine image screening identified potential duplication among the panels in Figure 2A. We understand that these images represent a time series. However, if the same sample is shown across multiple panels, this should be explicitly stated in the figure legend to avoid potential confusion for readers.

12. Please provide source data for all main manuscript figures. You should have received a separate email on March 24 with instructions for preparing, organizing, and uploading the source data files together with your revised manuscript. If you did not receive this email, please let us know and we will be happy to send it again.

Additional information on source data and instruction on how to label the files are available <
https://link.springer.com/journal/44320/submission-guidelines#cms-Source-data >

13. Please address the following issues in figure legends:
- Please define the annotated p values **** as well as provide the exact p-values for the same in the legend of figure EV3; as appropriate.
-Please note that the exact p values are not provided in the legends of figures 2E; 3B,C,I; 4C,M,N.
-Please indicate the statistical test used for data analysis in the legends of figures 4K-N.
- Please note that information related to n is missing in thelegends of figures 3I; 4C-F,P,Q,R; 7B,F,I,J,K; EV-3; EV-4.
- Please note that the error bars are not defined in the legends of figures 2B,J,K; 3B,C,I; 4C-F,P,Q,R; 7B,F,I,J,K; EV-3.
-Please note that the measure of center for the error bars needs to be defined in the legends of figures 7D.
-Please note that the yellow arrows and yellow dotted circles are not defined in the legend of 2A. This needs to be rectified.

14. Sections need to be named and the order should be corrected: Title page - Abstract - Introduction - Results - Discussion - Methods - Data Availability - Acknowledgements - Disclosure and Competing Interests Statement - References - Figure Legends - Table(s) - Expanded View Figure Legends.

Use the following link to submit your revised paper:

https://msb.msubmit.net/cgi-bin/main.plex

As a matter of course, please make sure that you have correctly followed the instructions for authors as given on the submission website.

Thank you for submitting this interesting paper to Molecular Systems Biology.

Kind regards,
Jingyi

Jingyi Hou, PhD
Senior Editor
Molecular Systems Biology

*** PLEASE NOTE *** As part of the EMBO Press transparent editorial process initiative (see our Editorial at https://dx.doi.org/10.1038/msb.2010.72 , Molecular Systems Biology will publish online a Review Process File to accompany accepted manuscripts. When preparing your letter of response, please be aware that in the event of acceptance, your cover letter/point-by-point document will be included as part of this File, which will be available to the scientific community. More information about this initiative is available in our Instructions to Authors. If you have any questions about this initiative, please contact the editorial office (msb@embo.org).

Reviewer #1:

The authors addressed my comments as requested and I found their answers convincing and responsive.

Reviewer #2:

This review focuses on the deep learning comments this reviewer made in the previous round. With the revisions made, the work is much more clear and is appreciated. The authors added a benchmark of the deep learning framework to show that it provided an added benefit are convincing and justify the complexity introduced, as well as improved the explanation of the technology and how it can generalize. Also, thank you for sharing the code! The code says data was deposited on Synapse - here a synapse ID would be helpful as the repository is not, by default, searchable. I have only one comment: The main text says the network has 11 layers and 23 total layers - is this a typo? Other than this confusion, the article is ready for publication.

Reviewer #3:

The authors have addressed the concerns of the prior review.

Response to Reviewer #2

We thank Reviewer 2 for comment and are glad that the revision has clarified previous concerns. We address each point below. Reviewer comments are shown in ***bold italic***, and our responses follow in regular text.

I have only one comment: The main text says the network has 11 layers and 23 total layers - is this a typo? Other than this confusion, the article is ready for publication.

We thank the reviewer for the comment. By convention, the layer number represents the number of trainable layers. The total layers, on the other hand, account for activation, input, output layers. To avoid the confusion, we have modified the text as follows to be consistent with the convention. The number of total layers is still available in the figures for readers for documentation and completeness.

“an 11-layer (23 total layers) deep neural network” -> “an 11-layer deep neural network”

21st Aug 2026
Manuscript number: MSB-2026-13666RR
Title: Decoding spatiotemporal fibrotic and cellular immunosuppression of T cells in live pancreatic cancer

Dear Paolo,

Thank you again for sending us your revised manuscript. We are now satisfied with the modifications made and I am pleased to inform you that your paper has been accepted for publication.

Your manuscript will be processed for publication by EMBO Press. It will be copy edited and you will receive page proofs prior to publication. Please note that you will be contacted by Springer Nature Author Services to complete licensing and payment information.

You may qualify for financial assistance for your publication charges - either via a Springer Nature fully open access agreement or an EMBO initiative. Check your eligibility: https://link.springer.com/journal/44320/how-to-publish-with-us

Should you be planning a Press Release on your article, please notify the production team via this form in order to coordinate publication and release dates: https://forms.cloud.microsoft/e/eH4A8JVP6g

If you have any questions, please do not hesitate to contact the Editorial Office. Thank you for your contribution to Molecular Systems Biology.

Sincerely,
Jingyi

Jingyi Hou, PhD
Senior Editor
Molecular Systems Biology

>>> Please note that it is Molecular Systems Biology policy for the transcript of the editorial process (containing referee reports and your response letter) to be published as an online supplement to each paper. If you do NOT want this, you will need to inform the Editorial Office via email immediately. More information is available here: https://link.springer.com/partners/embo-press/editorial-policies#Peer%20review

EMBO Press Author Checklist

Corresponding Author Name: Paolo Provenzano
Journal Submitted to: Molecular Systems Biology
Manuscript Number: MSB-2026-13666

USEFUL LINKS FOR COMPLETING THIS FORM
The EMBO Journal - Author Guidelines
EMBO Reports - Author Guidelines
Molecular Systems Biology - Author Guidelines
EMBO Molecular Medicine - Author Guidelines

Reporting Checklist for Life Science Articles (updated January
This checklist is adapted from Materials Design Analysis Reporting (MDAR) Checklist for Authors. MDAR establishes a minimum set of requirements in transparent reporting in the life sciences (see Statement of Task: 10.31222/osf.io/9sm4x). Please follow the journal's guidelines in preparing your
Please note that a copy of this checklist will be published alongside your article.

Abridged guidelines for figures
1. Data
The data shown in figures should satisfy the following conditions:
- the data were obtained and processed according to the field's best practice and are presented to reflect the results of the experiments in an accurate and unbiased manner.
- ideally, figure panels should include only measurements that are directly comparable to each other and obtained with the same assay.
- plots include clearly labeled error bars for independent experiments and sample sizes. Unless justified, error bars should not be shown for technical
- if n<5, the individual data points from each experiment should be plotted. Any statistical test employed should be justified.
- Source Data should be included to report the data underlying figures according to the guidelines set out in the authorship guidelines on Data

2. Captions
Each figure caption should contain the following information, for each panel where they are relevant:
- a specification of the experimental system investigated (eg cell line, species name).
- the assay(s) and method(s) used to carry out the reported observations and measurements.
- an explicit mention of the biological and chemical entity(ies) that are being measured.
- an explicit mention of the biological and chemical entity(ies) that are altered/varied/perturbed in a controlled manner.
- the exact sample size (n) for each experimental group/condition, given as a number, not a range;
- a description of the sample collection allowing the reader to understand whether the samples represent technical or biological replicates (including how many animals, litters, cultures, etc.).
- a statement of how many times the experiment shown was independently replicated in the laboratory.
- definitions of statistical methods and measures:
  - common tests, such as t-test (please specify whether paired vs. unpaired), simple χ2 tests, Wilcoxon and Mann-Whitney tests, can be unambiguously identified by name only, but more complex techniques should be described in the methods section;
  - are tests one-sided or two-sided?
  - are there adjustments for multiple comparisons?
  - exact statistical test results, e.g., P values = x but not P values < x;
  - definition of 'center values' as median or average;
  - definition of error bars as s.d. or s.e.m.

Please complete ALL of the questions below.
Select "Not Applicable" only when the requested information is not relevant for your study.

Materials

Newly Created Materials	Information included in the manuscript?	In which section is the information available? (Reagents and Tools Table, Materials and Methods, Figures, Data Availability Section)
New materials and reagents need to be available; do any restrictions apply?	Not Applicable	

Antibodies	Information included in the manuscript?	In which section is the information available? (Reagents and Tools Table, Materials and Methods, Figures, Data Availability Section)
For **antibodies** provide the following information:<br>- Commercial antibodies: RRID (if possible) or supplier name, catalogue number and or/clone number<br>- Non-commercial: RRID or citation	Yes	Methods

DNA and RNA sequences	Information included in the manuscript?	In which section is the information available? (Reagents and Tools Table, Materials and Methods, Figures, Data Availability Section)
**Short novel DNA or RNA including primers, probes**: provide the sequences.	Not Applicable	

Cell materials	Information included in the manuscript?	In which section is the information available? (Reagents and Tools Table, Materials and Methods, Figures, Data Availability Section)
**Cell lines:** Provide species information, strain. Provide accession number in repository **OR** supplier name, catalog number, clone number, and/**OR** RRID.	Not Applicable	
**Primary cultures:** Provide species, strain, sex of origin, genetic modification status.	Yes	Methods
Report if the cell lines were recently **authenticated** (e.g., by STR profiling) and tested for mycoplasma contamination.	Not Applicable	

Experimental animals	Information included in the manuscript?	In which section is the information available? (Reagents and Tools Table, Materials and Methods, Figures, Data Availability Section)
**Laboratory animals or Model organisms:** Provide species, strain, sex, age, genetic modification status. Provide accession number in repository **OR** supplier name, catalog number, clone number, **OR** RRID.	Yes	Methods
**Animal observed in or captured from the field:** Provide species, sex, and age where possible.	Not Applicable	
Please detail **housing and husbandry conditions**.	Yes	Methods

Plants and microbes	Information included in the manuscript?	In which section is the information available? (Reagents and Tools Table, Materials and Methods, Figures, Data Availability Section)
**Plants:** provide species and strain, ecotype and cultivar where relevant, unique accession number if available, and source (including location for collected wild specimens).	Not Applicable	
**Microbes:** provide species and strain, unique accession number if available, and source.	Not Applicable	

Human research participants	Information included in the manuscript?	In which section is the information available? (Reagents and Tools Table, Materials and Methods, Figures, Data Availability Section)
If collected and within the bounds of privacy constraints report on age, sex and gender or ethnicity for all study participants.	Not Applicable	

Core facilities	Information included in the manuscript?	In which section is the information available? (Reagents and Tools Table, Materials and Methods, Figures, Data Availability Section)
If your work benefited from core facilities, was their service mentioned in the acknowledgments section?	Not Applicable	

Design

Study protocol	Information included in the manuscript?	In which section is the information available? (Reagents and Tools Table, Materials and Methods, Figures, Data Availability Section)
If study protocol has been **pre-registered, provide DOI in the manuscript**. For clinical trials, provide the trial registration number **OR** cite DOI.	Not Applicable	
Report the **clinical trial registration number** (at ClinicalTrials.gov or equivalent), where applicable.	Not Applicable	

Laboratory protocol	Information included in the manuscript?	In which section is the information available? (Reagents and Tools Table, Materials and Methods, Figures, Data Availability Section)
Provide DOI OR other citation details if **external detailed step-by-step protocols** are available.	Yes	Methods

Experimental study design and statistics	Information included in the manuscript?	In which section is the information available? (Reagents and Tools Table, Materials and Methods, Figures, Data Availability Section)
Include a statement about **sample size** estimate even if no statistical methods were used.	Not Applicable	
Were any steps taken to minimize the effects of subjective bias when allocating animals/samples to treatment (e.g. **randomization procedure**)? If yes, have they been described?	Yes	Methods
Include a statement about **blinding** even if no blinding was done.	Not Applicable	
Describe **inclusion/exclusion criteria** if samples or animals were excluded from the analysis. Were the criteria pre-established?<br><br>If sample or data points were omitted from analysis, report if this was due to attrition or intentional exclusion and provide justification.	Not Applicable	
For every figure, are **statistical tests** justified as appropriate? Do the data meet the assumptions of the tests (e.g., normal distribution)? Describe any methods used to assess it. Is there an estimate of variation within each group of data? **Is** the variance similar between the groups that are being statistically compared?	Not Applicable	

Sample definition and in-laboratory replication	Information included in the manuscript?	In which section is the information available? (Reagents and Tools Table, Materials and Methods, Figures, Data Availability Section)
In the figure legends: state number of times the experiment was **replicated** in laboratory.	Yes	Figures
In the figure legends: define whether data describe **technical or biological replicates**.	Not Applicable	

Ethics

Ethics	Information included in the manuscript?	In which section is the information available? (Reagents and Tools Table, Materials and Methods, Figures, Data Availability Section)
Studies involving **human participants**: State details of **authority granting ethics approval** (IRB or equivalent committee(s), provide reference number for approval.	Not Applicable	
Studies involving **human participants**: Include a statement confirming that **informed consent** was obtained from all subjects and that the experiments conformed to the principles set out in the WMA Declaration of Helsinki and the Department of Health and Human Services Belmont Report.	Not Applicable	
Studies involving **human participants:** For publication of **patient photos**, include a statement confirming that consent to publish was obtained.	Not Applicable	
Studies involving experimental **animals**: State details of **authority granting ethics approval** (IRB or equivalent committee(s), provide reference number for approval. Include a statement of compliance with ethical regulations.	Yes	Methods
Studies involving **specimen and field samples:** State if relevant **permits** obtained, provide details of authority approving study; if none were required, explain why.	Not Applicable	

Dual Use Research of Concern (DURC)	Information included in the manuscript?	In which section is the information available? (Reagents and Tools Table, Materials and Methods, Figures, Data Availability Section)
Could your study fall under dual use research restrictions? Please check biosecurity documents and list of **select agents and toxins** (CDC): https://www.selectagents.gov/sat/list.htm	Not Applicable	
If you used a select agent, is the security level of the lab appropriate and reported in the manuscript?	Not Applicable	
If a study is subject to dual use research of concern regulations, is the name of the **authority granting approval and reference number** for the regulatory approval provided in the manuscript?	Not Applicable	

Reporting

The MDAR framework recommends adoption of discipline-specific guidelines, established and endorsed through community initiatives. Journals have their own policy about requiring specific guidelines and recommendations to complement MDAR.

Adherence to community standards	Information included in the manuscript?	In which section is the information available? (Reagents and Tools Table, Materials and Methods, Figures, Data Availability Section)
State if relevant guidelines or checklists (e.g., **ICMJE, MIBBI, ARRIVE, PRISMA**) have been followed or provided.	Not Applicable	
For **tumor marker prognostic studies,** we recommend that you follow the **REMARK** reporting guidelines (see link list at top right). See author guidelines, under 'Reporting Guidelines'. Please confirm you have followed these guidelines.	Not Applicable	
For **phase II and III randomized controlled trials**, please refer to the **CONSORT** flow diagram (see link list at top right) and submit the CONSORT checklist (see link list at top right) with your submission. See author guidelines, under 'Reporting Guidelines'. Please confirm you have submitted this list.	Not Applicable	

Data Availability

Data availability	Information included in the manuscript?	In which section is the information available? (Reagents and Tools Table, Materials and Methods, Figures, Data Availability Section)
Have **primary datasets** been deposited according to the journal's guidelines (see 'Data Deposition' section) and the respective accession numbers provided in the Data Availability Section?	Yes	Data availability
Were **human clinical and genomic datasets** deposited in a public access-controlled repository in accordance to ethical obligations to the patients and to the applicable consent agreement?	Not Applicable	
Are **computational models** that are central and integral to a study available without restrictions in a machine-readable form? Were the relevant accession numbers or links provided?	Yes	Data availability
If publicly available data were reused, provide the respective **data citations in the reference list**.	Not Applicable	
