# Supplementary material for: Decoding spatiotemporal fibrotic and cellular immunosuppression of therapeutic T cells in live pancreatic ductal adenocarcinoma
Source: Mol Syst Biol. 2026 Sep 10;22(10):1508–30. doi: 10.1038/s44320-026-00243-4 (PMC13639077; doi:10.1038/s44320-026-00243-4)
Supplement: Supplementary file 5 [file 44320_2026_243_MOESM4_ESM.zip › Movie EV1.docx]

**Video EV1** - MPLSM of CD8+ T cell migration in *KPCT* tumor slices showing heterogeneous T cell behaviors modulated by contact guidance cues from ECM architectures.
