# Supplementary material for: Decoding spatiotemporal fibrotic and cellular immunosuppression of therapeutic T cells in live pancreatic ductal adenocarcinoma
Source: Mol Syst Biol. 2026 Sep 10;22(10):1508–30. doi: 10.1038/s44320-026-00243-4 (PMC13639077; doi:10.1038/s44320-026-00243-4)
Supplement: Supplementary file 6 [file 44320_2026_243_MOESM5_ESM.zip › Movie EV2.docx]

**Video EV2** - MPLSM of meso-TCR T cell migration in *KPCT* tumor slices with labeling of CD11b+ myeloid cells showing T cell interactions in collagen-myeloid complexes.
